# Functional metagenomics of the thioredoxin superfamily

**DOI:** 10.1074/jbc.RA120.016350

**Published:** 2021-01-14

**Authors:** Sebastian Nilewski, Marharyta Varatnitskaya, Thorsten Masuch, Anna Kusnezowa, Manuela Gellert, Anne F. Baumann, Natalie Lupilov, Witali Kusnezow, Markus-Hermann Koch, Martin Eisenacher, Mehmet Berkmen, Christopher H. Lillig, Lars I. Leichert

**Affiliations:** 1 Institute of Biochemistry and Pathobiochemistry – Microbial Biochemistry, Ruhr-Universität Bochum, Bochum, Germany; 2 Protein Expression and Modification Division, New England Biolabs, Ipswich, Massachusetts, USA; 3 Institute for Medical Biochemistry and Molecular Biology, Universität Greifswald, Greifswald, Germany; 4 Medizinisches Proteom-Center, Ruhr-Universität Bochum, Bochum, Germany

**Keywords:** thioredoxin, *Escherichia coli* (*E. coli*), thiol, reductase, oxidase, protein disulfide isomerase, TrxA, DsbA, DsbC, thiol-disulfide oxidoreductase, AMS, 4-acetamido-4'-maleimidylstilbene-2,2'-disulfonic acid, CXXC, Cys-X-X-Cys, GOS, Global Ocean Sampling, Ni–NTA, nickel–nitrilotriacetic acid, PAPS, 3'-phosphoadenosine-5'-phosphosulfate, pCC, plasmid for cytoplasmic complementation, PDIs, protein disulfide isomerases, PNK, polynucleotide kinase, pOE, plasmid for overexpression, pPC, plasmid for periplasmic complementation, TCA, trichloroacetic acid, TEV, tobacco etch virus, vtPA, truncated version of human tissue plasminogen activator

## Abstract

Environmental sequence data of microbial communities now makes up the majority of public genomic information. The assignment of a function to sequences from these metagenomic sources is challenging because organisms associated with the data are often uncharacterized and not cultivable. To overcome these challenges, we created a rationally designed expression library of metagenomic proteins covering the sequence space of the thioredoxin superfamily. This library of 100 individual proteins represents more than 22,000 thioredoxins found in the Global Ocean Sampling data set. We screened this library for the functional rescue of *Escherichia coli* mutants lacking the thioredoxin-type reductase (Δ*trxA*), isomerase (Δ*dsbC*), or oxidase (Δ*dsbA*). We were able to assign functions to more than a quarter of our representative proteins. The *in vivo* function of a given representative could not be predicted by phylogenetic relation but did correlate with the predicted isoelectric surface potential of the protein. Selected proteins were then purified, and we determined their activity using a standard insulin reduction assay and measured their redox potential. An unexpected gel shift of protein E5 during the redox potential determination revealed a redox cycle distinct from that of typical thioredoxin-superfamily oxidoreductases. Instead of the intramolecular disulfide bond formation typical for thioredoxins, this protein forms an intermolecular disulfide between the attacking cysteines of two separate subunits during its catalytic cycle. Our functional metagenomic approach proved not only useful to assign *in vivo* functions to representatives of thousands of proteins but also uncovered a novel reaction mechanism in a seemingly well-known protein superfamily.

The thioredoxin superfamily (1) is an ancient protein family. It is assumed that the first thioredoxins arose ∼4 billion years ago, close to the first occurrence of life on planet earth (2). The active site of thioredoxin-like proteins consists of a Cys-X-X-Cys (CXXC) motif located at the N terminus of an alpha helix (see Refs. (3, 4) for comprehensive reviews). In a redox reaction, the side chains of the two cysteines in this active site motif can switch from the free thiol (SH) form to a disulfide bond (S–S) and/or back and hence donate or accept electrons. The biological function of thioredoxin-like proteins is determined by the preference of the active site to either accept or donate electrons and also depends on the cellular redox context. The majority of thioredoxin superfamily members can be categorized into three major functional groups: thiol-disulfide reductases, which donate electrons in reduction reactions, oxidases, which accept electrons and oxidize their substrates, or isomerases, which rearrange disulfide bonds in substrate proteins. The thioredoxin fold (5) is highly versatile, and thus, members of the thioredoxin superfamily can have highly specialized functions, including reductases for specific substrates, for example, arsenate reductase (6). Some proteins with thioredoxin fold have further evolved to functions completely unrelated to redox reactions and do not even require a CXXC motif, such as calsequestrin (7).

While the biological function of a thioredoxin protein seems to be based on the redox potential of the hallmark CXXC motif, the activity toward substrates, and the subcellular context of the redox partners (8), the structure–function relationship in this protein class is not fully understood and hard to conjecture. Even with extensive structural information, the prediction of the redox potential of thioredoxins is challenging (9), and, to our knowledge, a prediction based on primary structure has not yet been attempted.

With the recent surge in the study of metagenomes, our knowledge about the primary structure of proteins in general and thioredoxins in particular has expanded substantially. In metagenomic projects, DNA from microbial communities is sequenced without the need for the isolation of individual organisms. Even in traditional genomics, the assignment of functions to protein-encoding genes is still a major bottleneck. In the metagenomic era, because the DNA sequenced is largely of microbial origin and the majority of microorganisms cannot be cultivated in the laboratory yet, functional assignment has become arguably even more challenging.

In this study, we attempt to overcome these challenges by utilizing the vast genetic information contained in metagenomes. For this, we devised a combination of bioinformatics, synthetic biology, and well-established *Escherichia coli* genetics to functionally characterize the thioredoxin superfamily. As the underlying data set for our study, we chose the Global Ocean Sampling (GOS) data set, one of the largest metagenomic data sets to date (10). In a first step, we used bioinformatics to identify all thioredoxin superfamily members in this data set. Then we categorized them into subfamilies, using a Markov clustering algorithm. We then identified 100 proteins representing these subfamilies and, using synthetic genes, expressed them in *E. coli*. Using knockout *E. coli* strains lacking thioredoxin-type reductases, oxidases, or isomerases, we could assign functions to these representatives based on complementation assays. Analyzing the predicted isoelectric surface potential, we were able to predict reductase function *post hoc*. We then purified and biochemically characterized selected thioredoxins, finding that neither *in vitro* activity nor redox potential correlates with function. Our data provide, to our knowledge, the biggest set of thioredoxin proteins with a known redox potential. One particular thioredoxin, E5, was shown to have an unprecedented mechanism of action, being an oligomer with an intermolecular disulfide bond forming between the attacking cysteines of two subunits during its catalytic cycle.

## Results and discussion

### Categorization of thioredoxin superfamily members from a metagenomic data set

In order to advance our understanding of the structure–function relationship of the thioredoxin superfamily, we wanted to categorize all thioredoxin superfamily members found within the GOS data set into the three major groups: reductases, oxidases, and isomerases. First, we selected all sequences from a nonredundant GOS data set that matched to any of the hidden Markov models of the Pfam thioredoxin clan (CL0172) with a score below 10^−5^ (11). Of these 22,860 matches, we removed all 18,187 sequences that did not include an unambiguous start methionine or a stop codon. These incomplete thioredoxin sequences were excluded, as it is not possible to synthesize a functional gene from these sequences. We further removed three sequences that did not contain cysteines at the active site found at the N terminus of the first alpha helix, reducing the set to a total of 4673 complete thioredoxins. We then used a Markov cluster algorithm to divide the thioredoxin set into subfamilies (12). The algorithm defined 49 clades, which ranged in size from 1 to 1064 sequences (see Table S1 for accession numbers).

**Figure 1:**
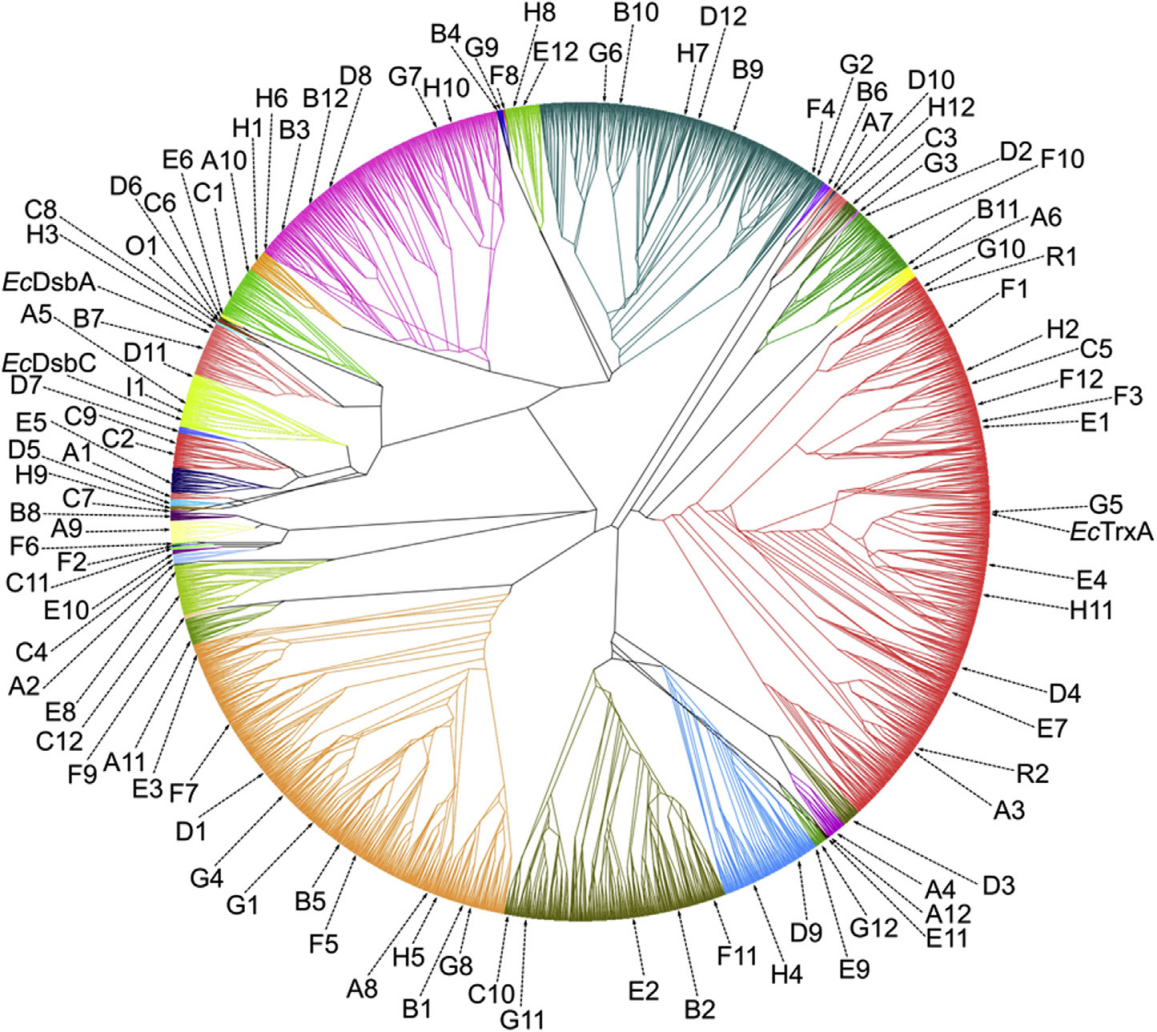
**Phylogenetic tree of all 4678 complete thioredoxin superfamily members found in the Global Ocean Sampling data set.** Different colors represent phylogenetic clades identified by Markov clustering. Representative sequences chosen for this study are indicated by *arrows*. See also Table S1 and Supporting Information S1.

Next, we created phylogenetic trees of these clusters and calculated from these trees the mean evolutionary distance for each individual sequence to all other sequences within the tree. This mean evolutionary distance essentially provided us with a measure for the relationship distance of any individual protein to all other proteins within a cluster. The protein most closely related to all other cluster members was defined as the representative of that cluster (Table S1). In exceptionally large clusters, we chose additional proteins with greater mean evolutionary distances to cover the whole diversity of those families (Fig. 1 and Table S1). In this way, we were able to select 100 individual proteins representing the totality of the thioredoxin universe in the GOS data set. Genes encoding these proteins were then codon optimized for *E. coli* and synthesized (see Supporting Information S1 for the protein sequences of the synthesized genes).

### Functional complementation assays

**Figure 2:**
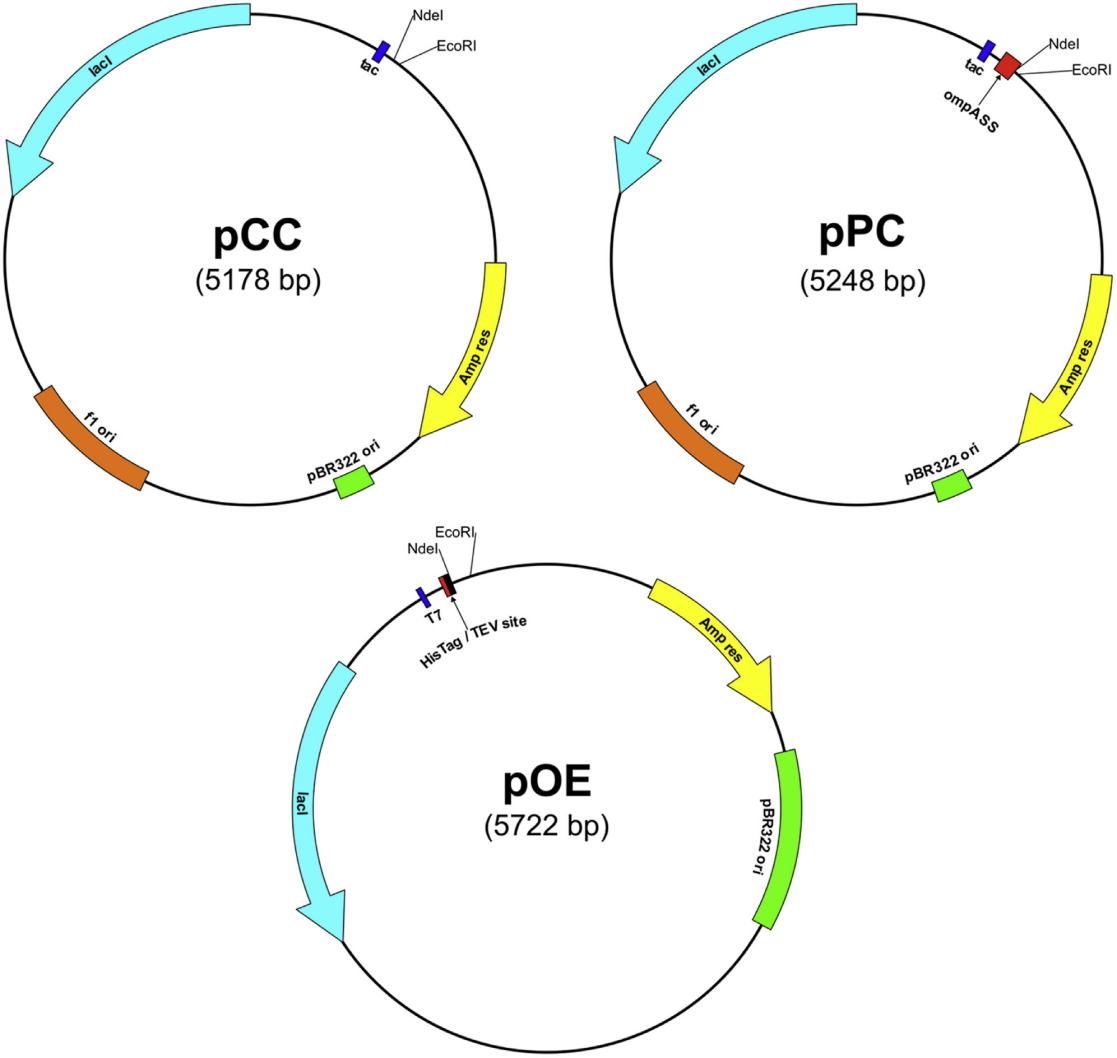
**Plasmids used in this study.** pCC (*p*lasmid for *C*ytoplasmic *C*omplementation) and pPC (*p*lasmid for *P*eriplasmic *C*omplementation) are derivatives of pTAC-MAT-Tag-2 (Sigma–Aldrich). pOE (*p*lasmid for *O*ver*E*xpression) is a derivative of pET-11a (Agilent). Thioredoxin superfamily members were inserted between *Nde*I (containing the original gene's start codon ATG) and *Eco*RI. Inserted genes contained a stop codon before *Eco*RI. For vector sequences, see also Supporting Information S2.

To assign functions to these 100 representative proteins, we developed a complementation system in *E. coli*. The three major functional groups of thioredoxin superfamily members are represented in *E. coli* by the proteins thioredoxin 1 (TrxA, a reductase), DsbA (an oxidase), and DsbC (an isomerase). Based on the phenotypes of mutants lacking these proteins, we established complementation assays with the help of a set of vectors that introduce the metagenomic thioredoxins into *E. coli* mutants. These vectors express one of the 100 synthetic genes in the cytoplasm (plasmid for cytoplasmic complementation [pCC]) or the periplasm of *E. coli* (plasmid for periplasmic complementation [pPC]), or express them with an N-terminal tobacco etch virus (TEV) protease-cleavable His_6_ tag for purification (plasmid for overexpression [pOE]) (Fig. 2, vector sequences: Supporting Information S2). The individual complementation assays were set up as follows:

### Reductase assay

**Figure 3:**
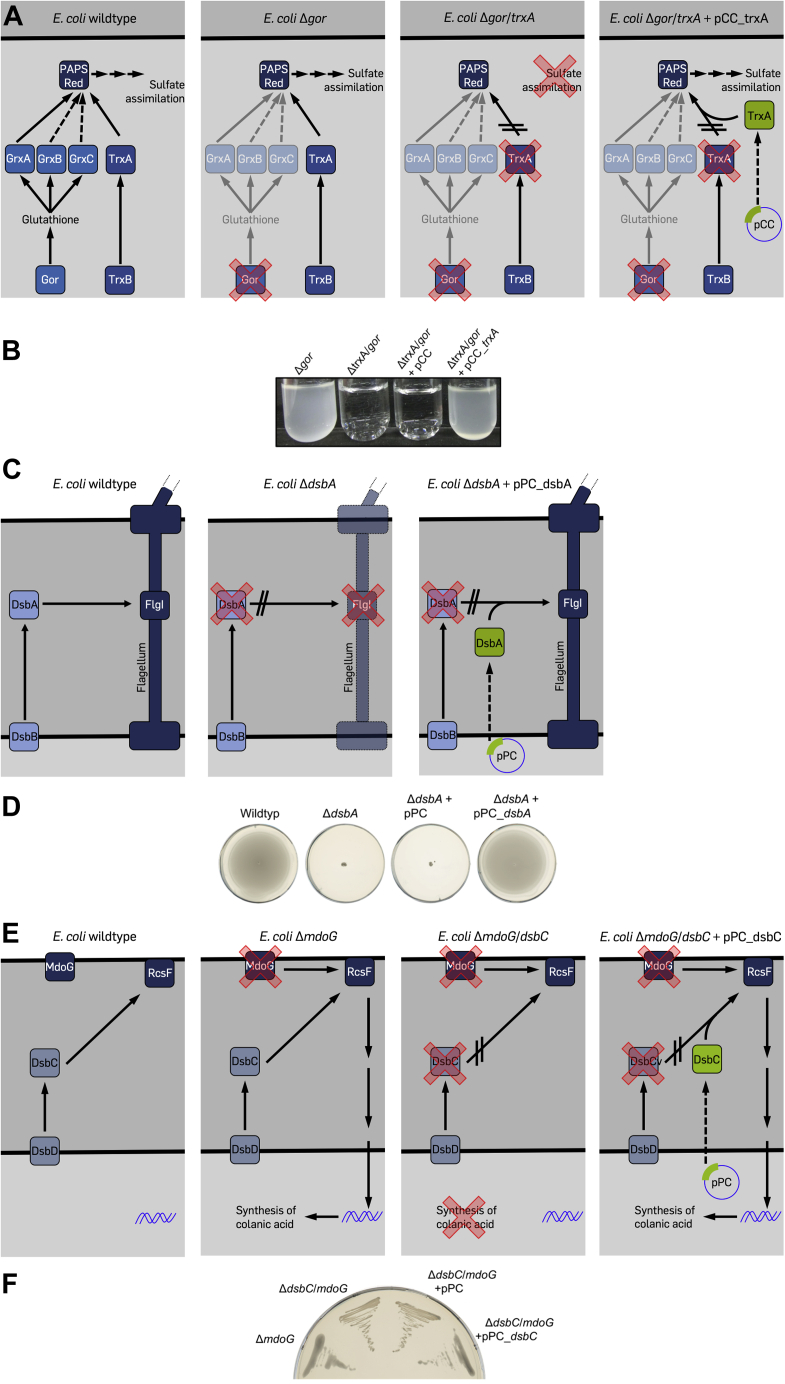
**Rescue of phenotypes of thioredoxin superfamily member knockouts in *Escherichia coli*.***A*, schematic of the *trxA* complementation assay. *E. coli* wildtype is able to grow on sulfate as the only sulfur source using 3'-phosphoadenosine-5'-phosphosulfate-reductase (PAPS red)-dependent sulfate assimilation. PAPS red itself is reduced by thioredoxin (TrxA) or one of the several glutaredoxins (GrxA, GrxB, and GrxC). The knockout mutant of glutathione oxidoreductase (*E. coli* Δ*gor*) effectively inactivates the glutaredoxins, but the strain is still able to grow on sulfate as the sole sulfur source because TrxA is sufficient to reduce PAPS red. A Δ*gor* Δ*trxA* double mutant (*E. coli* Δ*gor*/*trxA*) is no longer able to reduce PAPS red. The introduction of *trxA* from a plasmid (pCC_trxA) restores PAPS red reduction. *B*, a Δ*gor*/*trxA* double mutant is not viable in minimal medium containing sulfate as the sole sulfur source. Expression of *E. coli* TrxA from plasmid pCC rescues the phenotype. *C*, *E. coli* wildtype expresses functional flagella containing an essential disulfide bond in the FlgI subunit of the flagellar motor. This disulfide bond is introduced by the thiol-disulfide oxidoreductase DsbA. A knockout mutant lacking DsbA (*E. coli* Δ*dsbA*) is lacking functional flagella. Reintroduction of DsbA from a plasmid (pPC_dsbA) restores structural disulfide bonds in FlgI. *D*, a Δ*dsbA* mutant is nonmotile. Expression of *E. coli* DsbA from plasmid pPC rescues the phenotype. *E*, *E. coli* wildtype is nonmucoid. A knockout in MdoG (*E. coli* Δ*mdoG*) leads to a mucoid phenotype, as RcsF senses envelope stress and initiates a signaling cascade resulting in the production of colanic acid. The correct disulfide connectivity in RcsF is dependent on the isomerase DsbC, thus a mutant lacking both MdoG and DsbC (*E. coli* Δ*mdoG*/*dsbC*) is nonmucoid. Expression of DsbC from a plasmid (pPC_dsbC) restores mucoidity in this double mutant. *F*, a Δ*mdoG*/*dsbC* double knock forms nonmucoid colonies. Expression of *E. coli* DsbC without export signal sequence (–SS) from plasmid pPC restores mucoidity.

**Figure 4:**
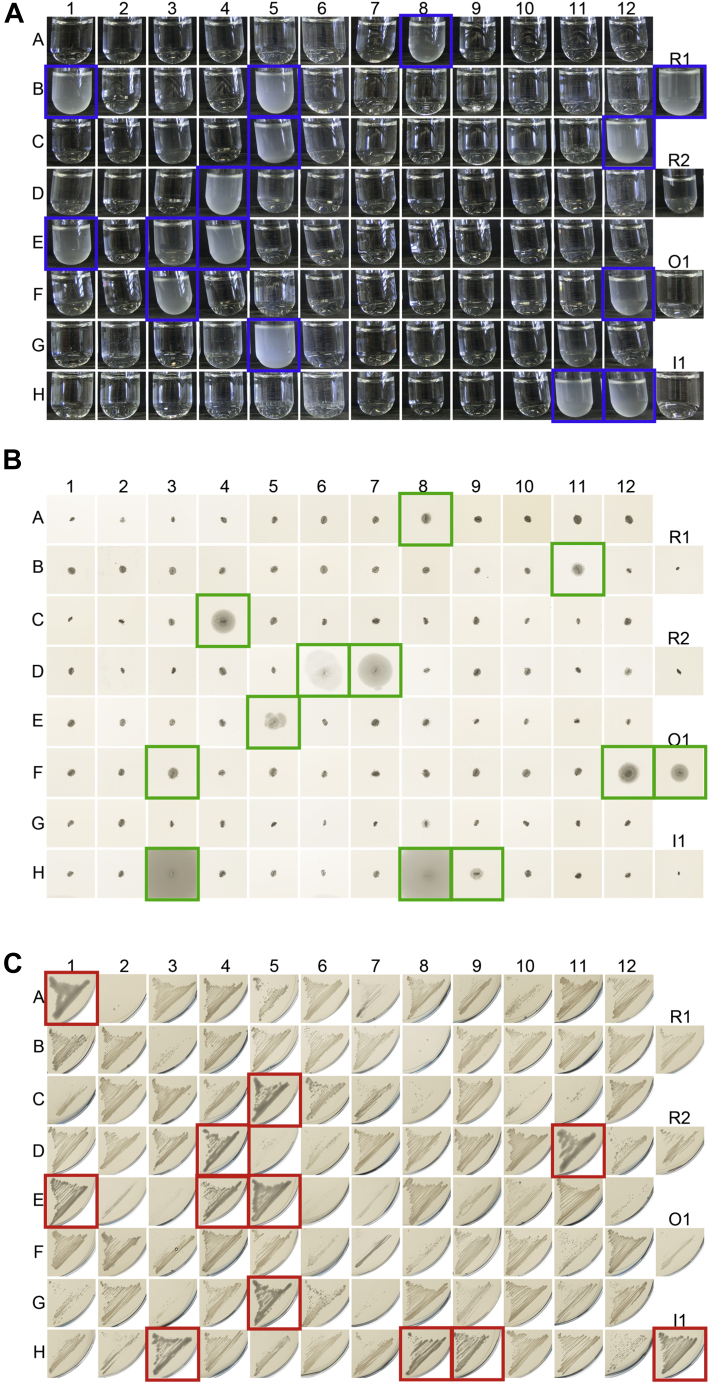
**Complementation assays for the assignment of functions to metagenomic thioredoxin superfamily members.***A*, growth phenotypes of Δ*gor* Δ*trxA* strains expressing 100 metagenomic thioredoxin superfamily members. A Δ*gor* Δ*trxA* strain of *Escherichia coli* is unable to grow in a minimal medium containing sulfate as the sole sulfur source (see Fig. 3*A* for a schematic). Only if a metagenomic reductase expressed from a plasmid acts on phosphoadenosine-5'-phosphosulfate reductase, the phenotype will be rescued. Metagenomic thioredoxin superfamily members rescuing the phenotype are highlighted in *blue*. *B*, motility phenotypes of Δ*dsbA* strains expressing 100 metagenomic thioredoxin superfamily members. A Δ*dsbA* strain is nonmotile (see Fig. 3*C* for a schematic). A metagenomic oxidase expressed from a plasmid could rescue the associated phenotype if it is able to introduce a structural disulfide in the bacterial flagella. Thioredoxin superfamily members restoring motility are highlighted in *green*. *C*, mucoidity of Δ *mdoG* Δ*dsbC* strains expressing 100 metagenomic thioredoxin superfamily members. A Δ*mdoG* Δ*dsbC* strain has a nonmucoid phenotype (see Fig. 3*E* for a schematic). Mucoidity could be restored by a metagenomic isomerase that can isomerize the correct disulfides in RcsF. Thioredoxin superfamily members rescuing RcsF function are highlighted in *red*. Representative results, all experiments were repeated at least three times.

*E. coli* TrxA is a cytoplasmic thiol-disulfide reductase that reduces disulfide bonds in several essential cellular reactions, among them a disulfide bond in 3'-phosphoadenosine-5'-phosphosulfate (PAPS) reductase that is generated during sulfate assimilation (13). The glutathione-dependent glutaredoxins can compensate for the lack of TrxA, but a Δ*gor* Δ*trxA* double mutant, which also lacks the ability to rereduce glutathione, is unable to grow in minimal medium containing sulfate as the sole sulfur source (14) (see Fig. 3*A* for a schematic overview). The reintroduction of a disulfide reductase such as *E. coli* TrxA or a metagenomic thioredoxin with reductase activity from a plasmid should rescue growth of this mutant in minimal medium. By expressing our 100 synthetic genes in this strain from the pCC plasmid, we identified 15 reductases in the set of metagenomic thioredoxin superfamily members (Figs. 3*B* and 4*A*).

### Oxidase assay

DsbA is a thiol-disulfide oxidase that introduces structural disulfides during oxidative folding in the periplasm of *E. coli* (15). Among the substrates of DsbA is FlgI, a protein essential for the formation of the bacterial flagellum. Thus, Δ*dsbA* null mutants are nonmotile (16) (see Fig. 3*C* for a schematic overview). A thiol oxidase expressed from a plasmid containing a periplasmic signal sequence should restore motility of that mutant. *E. coli* DsbA is able to restore that phenotype, as are 12 representative metagenomic thioredoxins (Figs. 3*D* and 4*B*).

### Isomerase assay

The isomerase DsbC rearranges disulfide bonds in periplasmic proteins (17). This function is particularly needed during folding of proteins containing nonconsecutive disulfide bonds in their structure (18). RcsF is a protein dependent on the activity of DsbC and is at the beginning of a cascade that activates colanic acid capsule synthesis upon perturbance of the envelope (19). A Δ*mdoG*(=*opgG*) mutant lacks osmoregulated periplasmic glucans and usually compensates this RcsF-dependently by an excess in colanic acid production (20). Colonies of Δ*mdoG* strains thus appear mucoid at low growth temperatures (21). However, because RcsF is inactive in the absence of DsbC, a Δ*mdoG ΔdsbC* double mutant has a regular colony shape (22) (see Fig. 3*E* for a schematic overview). A disulfide isomerase expressed from a plasmid and exported into the periplasm should lead to a mucoid colony shape in this mutant. In addition to *E. coli* DsbC, 12 metagenomic representative thioredoxins led to a restoration of the mucoid phenotype of this strain (Figs. 3*F* and 4*C*).

### 27 out of 100 thioredoxins show an *in vivo* function

**Figure 5:**
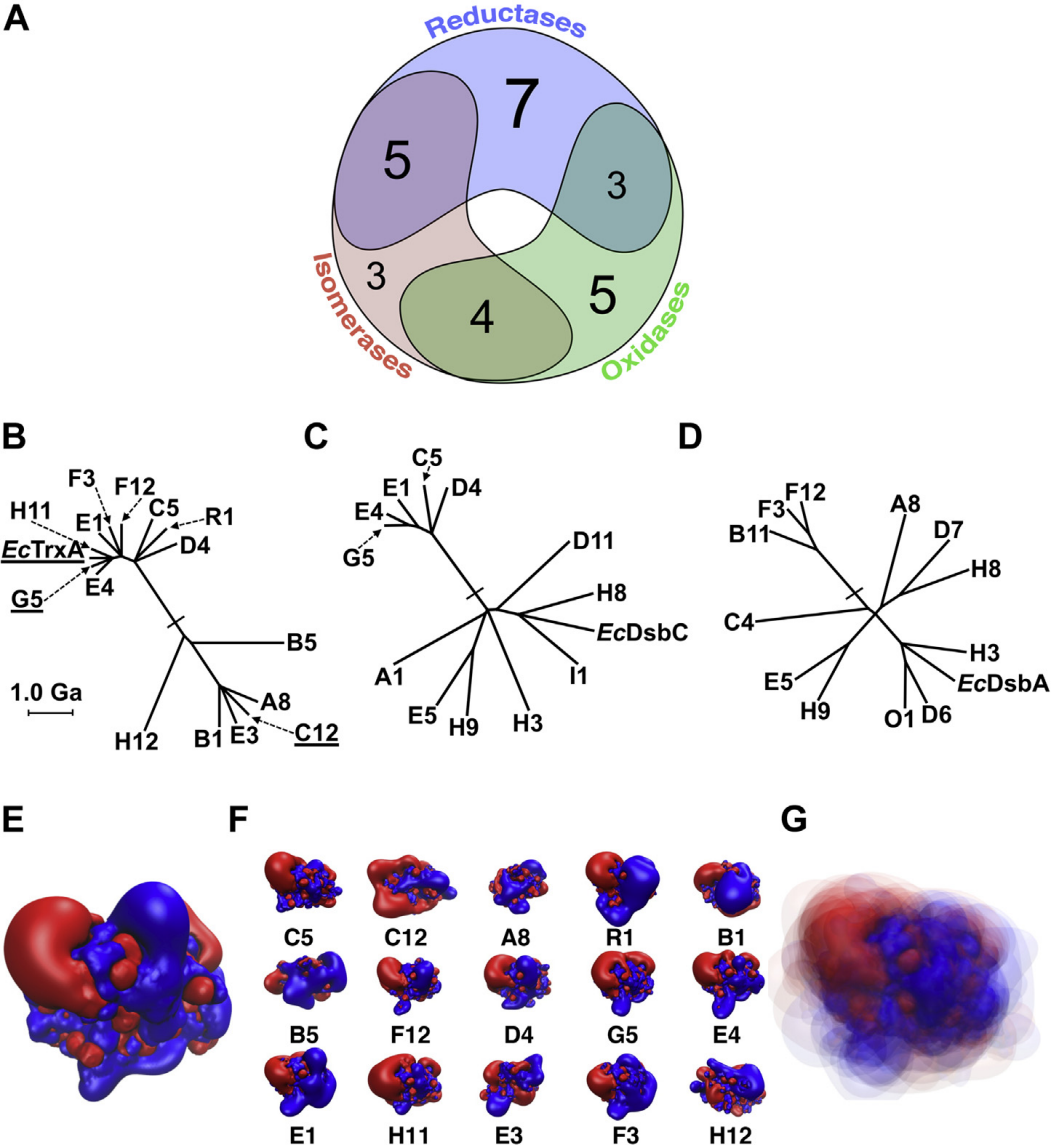
**Relation of assigned function with phylogeny of thioredoxin superfamily members and the isosurfaces of the electrostatic potential of reductases.***A*, 27 (out of 100) thioredoxin superfamily members were assigned a function based on our complementation assays. Twelve superfamily members rescued more than one phenotype. *B*–*D*, phylogenetic trees and evolutionary distance of *Escherichia coli* thioredoxins and their metagenomic counterparts. *B*, metagenomic reductases. *C*, metagenomic isomerases. *D*, metagenomic oxidases. See also Figure 1 for how these proteins relate to each other and to the other thioredoxins identified in the Global Ocean Sampling data set. The presumed root of the tree is marked by a *bar*. Alignments of the metagenomic reductases, oxidases, and isomerases can be found in Supporting Information S3. *E*, isoelectric surface charge of *E. coli* TrxA. *F*, isosurfaces of the electrostatic potential based on predicted structures of metagenomic reductases. Orientation of the proteins is identical based on the structural alignment with *E. coli* TrxA's structure. *G*, consensus isosurfaces of all metagenomic reductases. For predicted isoelectric surfaces of all metagenomic thioredoxins, see Fig. S1. For predicted isoelectric surfaces of metagenomic oxidases and isomerases, see Fig. S2.

**Figure 6:**
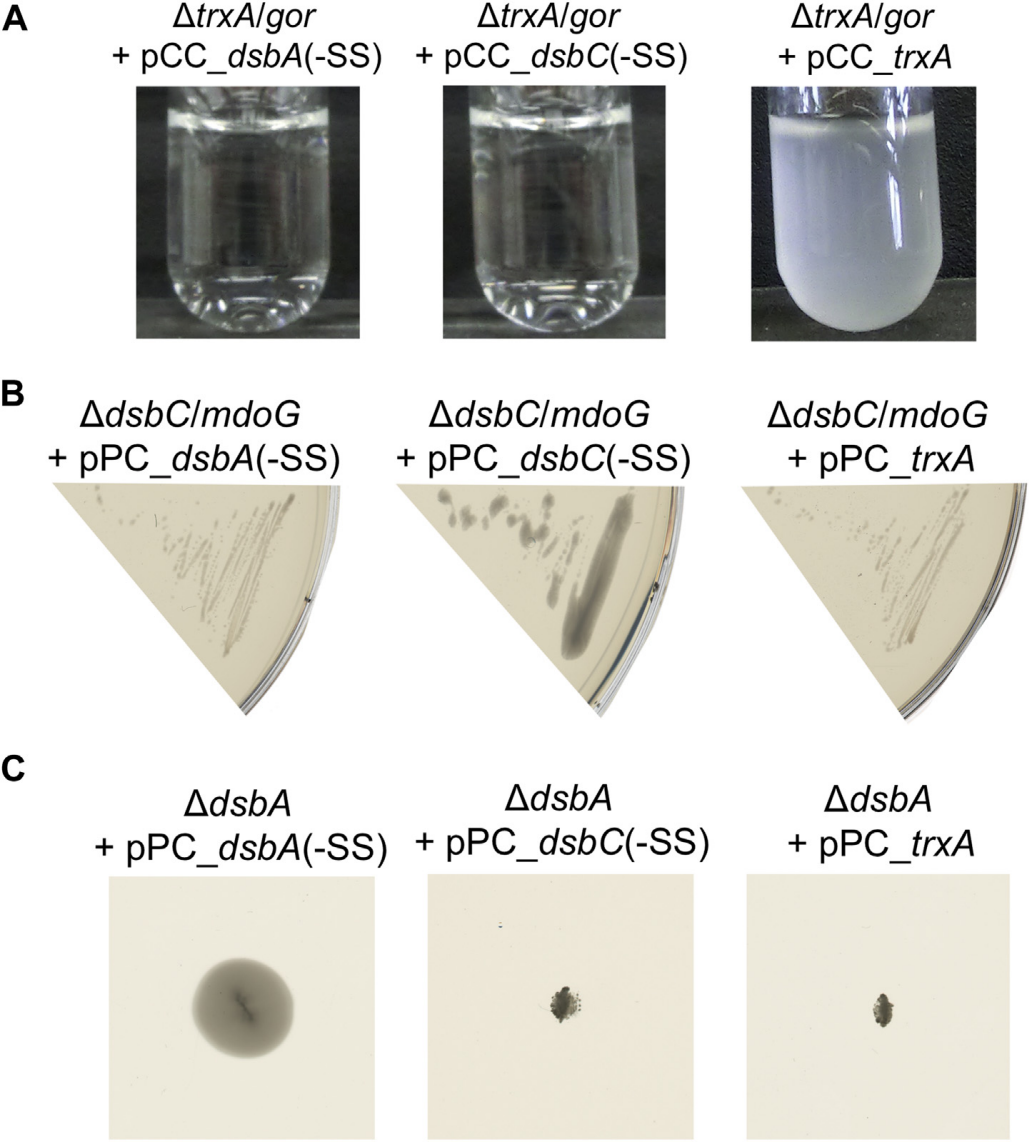
***Escherichia coli* TrxA (*A*), DsbC (*B*), and DsbA (*C*) only rescue their respective phenotype.** No overlapping function was detected in the selected assays. The native signal sequence for periplasmic export was removed from DsbC and DsbA (–SS).

With these three assays at hand, we were able to assign a function to 27 of our 100 thioredoxin superfamily members (Fig. 4). Based on the high-throughput nature of our assay, we cannot conclude that the other 73 proteins are inactive, as the probability of false negatives in such an approach is high. Twelve of the metagenomic thioredoxins showed overlapping activities, that is, they rescued more than one phenotype (Fig. 5*A*). The natural propensity of thioredoxin superfamily members to be functionally promiscuous, especially with regard to isomerase and oxidase activity, is already notable in eukaryotes, where those functions are achieved by a single protein (protein disulfide isomerase [PDI]). The observed promiscuity, however, was in contrast to their *E. coli* counterparts, which were exclusively able to rescue their own phenotype (Fig. 6).

### A thioredoxin's *in vivo* function is linked to its isoelectric surface potential

The overlapping activity of some thioredoxins already demonstrated the high plasticity of this protein family, although this promiscuity might in part be induced by the artificiality of our assay, such as unnatural high expression levels. To elucidate the connection between functionality and protein evolution, we then determined the phylogenetic relationship of the functionally complementing thioredoxins to their *E. coli* counterparts (Fig. 5, *B*–*D*; see also Fig. 1). Our set of 100 thioredoxin superfamily members contained a number of canonical folds and active sites typical for thioredoxins, glutaredoxins, glutathione-*S*-transferases, NrdH glutaredoxin-like proteins, arsenate reductases, PDIs, peroxiredoxins, and glutathione peroxidases (Table S2). Of those, only thioredoxin, glutaredoxin, PDI, and Dsb family members showed activity in our complementation assay. However, the wide diversity we found among those families and the fact that the fold did not necessarily align with the observed function suggested that phylogenetic relation is not a good predictor for thioredoxin function. Similarly, an analysis of the secondary structure based on structural predictions suggested that thioredoxins can share the same *in vivo* function, although their primary and secondary structure have substantially diverged, while presumably structurally similar thioredoxins can have opposing functions (see Supporting Information S4). Dissatisfied with our inability to predict the function of a thioredoxin based on a phylogenetic or a simplistic structure analysis, we then calculated the isoelectric surface potential (isosurfaces) of these proteins (Fig. S1). These results suggested that electrostatic fields emanating from the proteins play a large role in the *in vivo* function of these proteins. The isoelectric surface potential of the 15 proteins functioning as reductases showed a striking similarity to that of *E. coli* TrxA (Fig. 5, *E*–*G*), suggesting that in the case of reductases the electrostatic interactions between the metagenomic proteins and their *E. coli* substrates are a determinant of the protein's *in vivo* function.

### Oxidoreductase activity of metagenomic thioredoxins

**Figure 7:**
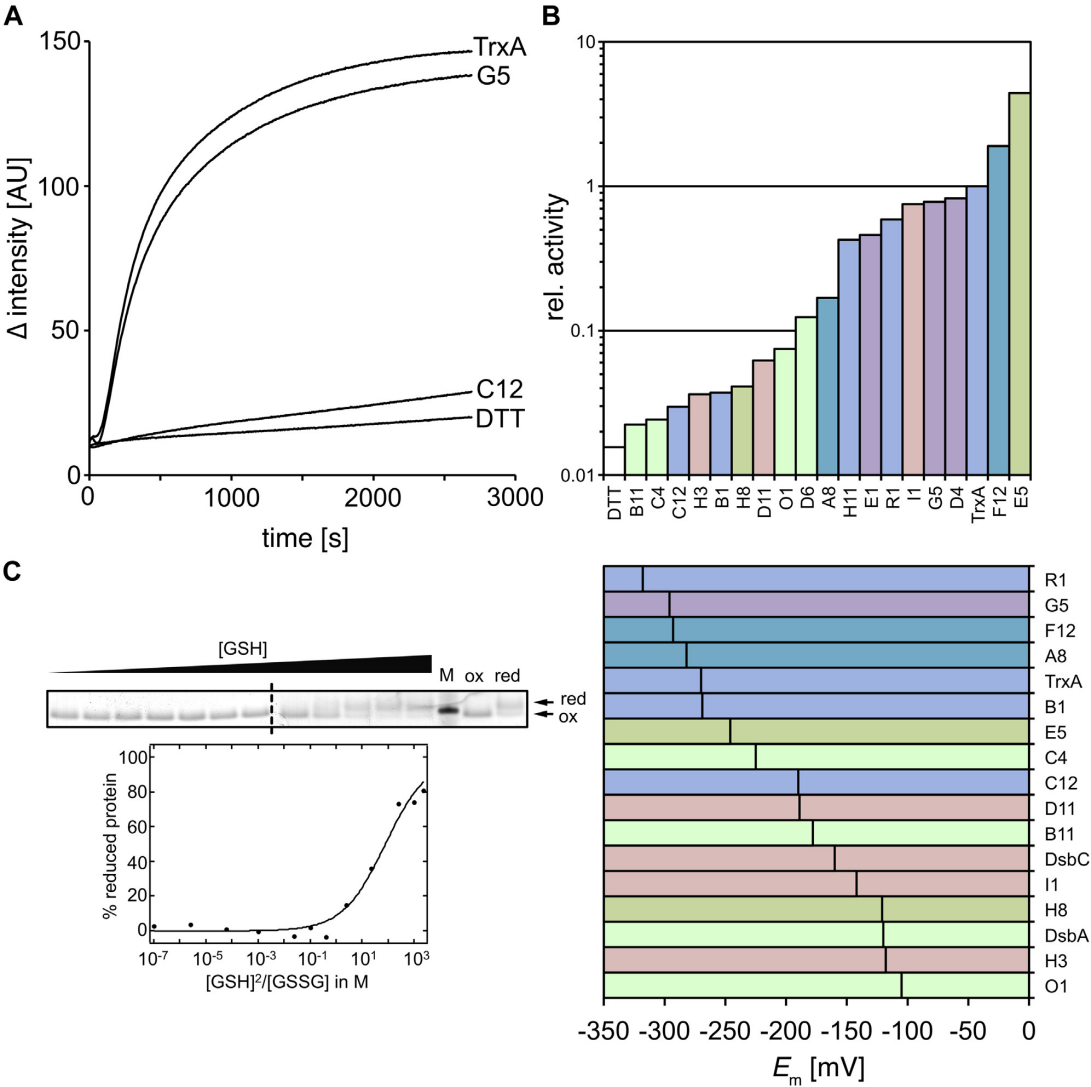
**Insulin reduction activity and midpoint redox potential of thioredoxin superfamily members.***A*, representative insulin reduction activity measurement of *Escherichia coli* TrxA and the metagenomic reductases G5 and C12, TrxA's phylogenetically most closely and distantly related thioredoxin superfamily members. *B*, relative insulin reduction activity of metagenomic thioredoxin superfamily members. Activity of *E. coli* TrxA was set to 1. Color coding according to function (*cf.*Fig. 5*A*). *C*, representative redox potential determination of metagenomic thioredoxin G5. *Arrows* labeled *red* and *ox* indicate the migration of the 4-acetamido-4'-maleimidylstilbene-2,2'-disulfonic acid–treated reduced and oxidized G5, respectively. Because of the limited number of available pockets on an SDS gel, samples were run on two separate gels. The *dashed* lane marks the splice border. *D*, standard redox potential E_0_ of metagenomic thioredoxin superfamily members. Color coding according to function (*cf.*Fig. 5*A*). M, marker.

To verify the *in vivo* activity derived from our complementation assays and to further substantiate the relationship between protein function and predicted isosurfaces, we purified and biochemically characterized selected thioredoxins from the set of 27 thioredoxins showing activity in *E. coli*. Thioredoxin superfamily members share the ability to catalyze the reduction of insulin by DTT (23). We used this classical insulin reduction assay to determine the activity of the respective metagenomic thioredoxins. In our assay, an FITC-labeled insulin was incubated with DTT and a metagenomic thioredoxin superfamily member was added as a catalyst (Fig. 7*A*). The reduction of insulin and separation of the two chains could be monitored spectrofluorometrically. The initial velocity was used as a measurement for activity. All active metagenomic thioredoxins examined showed insulin reduction activity. However, the activity of any given metagenomic thioredoxin did not correlate with its *in vivo* function (Fig. 7*B*).

### The metagenomic thioredoxins span a wide range of redox potentials

In addition to the kinetic parameter of insulin reduction activity, the standard redox potential is an important thermodynamic factor for thioredoxin function. The redox potential essentially determines the possible direction of the electron flow between partners in a redox network. In *E. coli*, the function of the three paradigmatic proteins TrxA (reductase with a redox potential of −270 mV), DsbA (oxidase with a redox potential of −120 mV), and DsbC (isomerase with a redox potential of −130 mV) correlates with their redox potential, with the reductase TrxA having the most reducing and the oxidase DsbA the most oxidizing active site (24, 25, 26). We, therefore, determined the redox potential of the selected thioredoxin superfamily members by incubation in redox buffers and subsequent 4-acetamido-4'-maleimidylstilbene-2,2'-disulfonic acid (AMS) labeling of free thiols. AMS chemically modifies reduced thiols and thus leads to retardation in a nonreducing SDS-PAGE. Based on this, we could determine the oxidized and reduced portions of a protein densitometrically and thus determine the midpoint redox potential (Fig. 7*C*). It is important to realize that the half-cell potential and the flow of electrons within the cell is not solely determined by the standard redox potential of the redox partners but often imposed by other systems: the NADPH and thioredoxin reductase transfer electrons and protons to oxidized TrxA; DsbB accepts electrons from reduced DsbA and donates them to ubiquinone; and DsbD keeps DsbC reduced in a Trx system-dependent manner. Consequently, some of the proteins with a midpoint redox potential lower than *E. coli* TrxA could also rescue the isomerase or oxidase phenotype *in vivo*, despite the substantial difference in their midpoint redox potential when compared with the *E. coli* protein they were complementing. The most extreme example was F12, which had a redox potential of −312 mV but could replace DsbA with a redox potential of −120 mV (Fig. 7*D*).

### E5, an unusual thioredoxin

**Figure 8:**
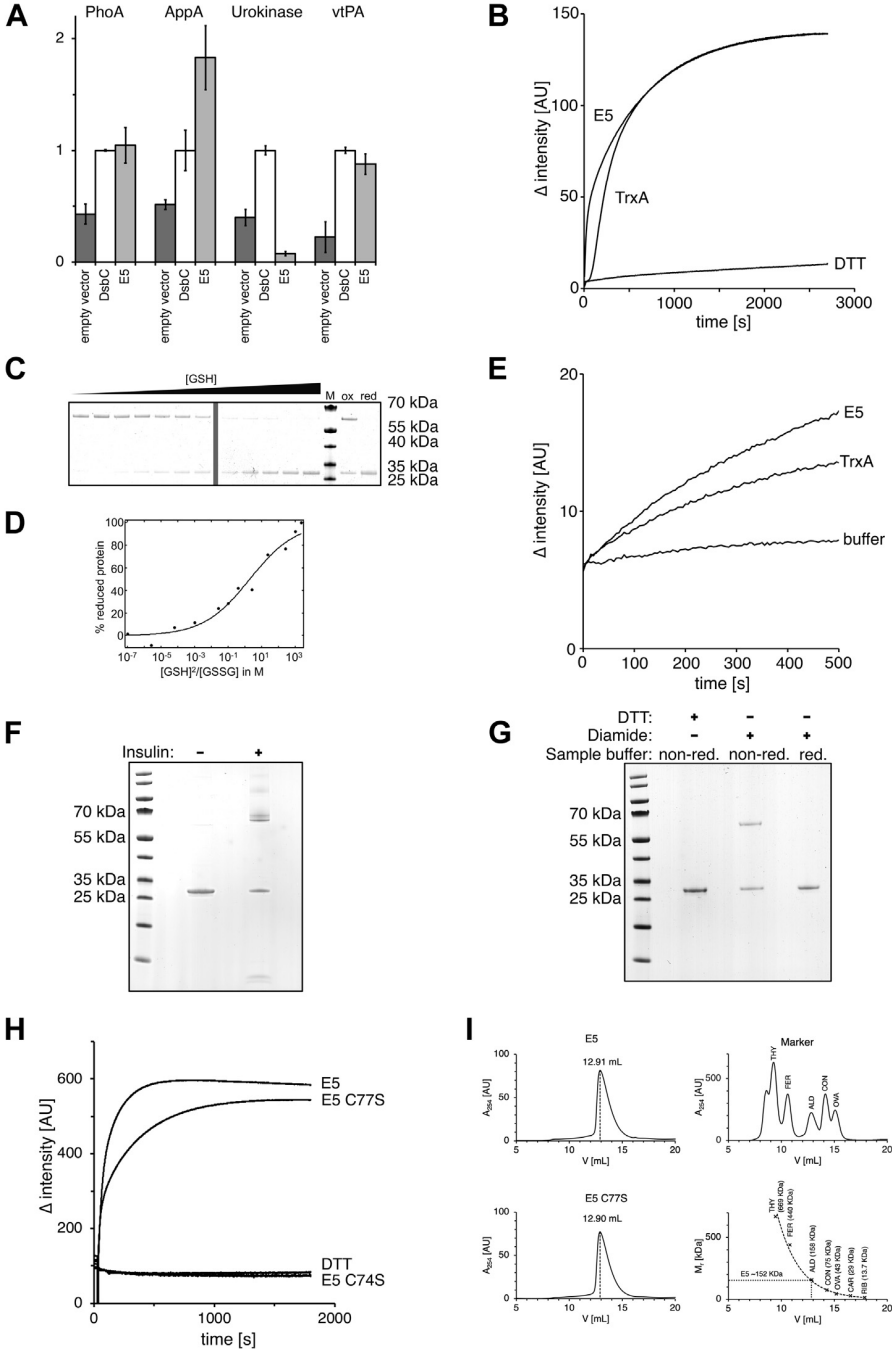
**Metagenomic thioredoxin E5 is a new i thioredoxin superfamily member with an unusual reaction mechanism.***A*, E5 is an effective disulfide isomerase in the commercial *Escherichia coli* SHUFFLE strain and can support oxidative folding of several proteins, including vtPA, which contains nine disulfide bonds when folded correctly. *B*, it shows higher initial velocity than *E. coli* TrxA in an insulin reduction assay. *C*, redox potential determination of E5 using 4-acetamido-4'-maleimidylstilbene-2,2'-disulfonic acid, as described for Figure 7*C*. When oxidized in a glutathione redox buffer, E5 forms disulfide-linked dimers, which can be observed in nonreducing SDS-PAGE. M, marker, MW in kDa indicated on the right, ox. and red.: fully oxidized and reduced E5, respectively. *D*, the redox potential can be calculated from the ratio of reduced and oxidized protein in a given glutathione redox buffer based on the Nernst equation. Based on these calculations, E5 has a standard redox potential of −246 mV, close to the redox potential of thioredoxin. *E*, reduced E5 and *E. coli* TrxA reduce insulin when no DTT for enzyme recycling is added. *F*, E5 protein oxidized in this reaction forms disulfide-linked dimers, which can be observed in a Coomassie-stained SDS-PAGE. Higher molecular weight species indicated by *arrows* are presumably insulin adducts to the E5 dimer and E5 multimers crosslinked by insulin. *G*, an E5 C77S mutant, which contains only one cysteine, still forms a disulfide-linked dimer when oxidized with diamide and (*H*) is still active in an insulin reduction assay, while an E5 C74S mutant is inactive. *I*, size exclusion choromatography of E5 and its C77S mutant suggests a molecular weight consistent approximately with a homohexamer (the calculated mass of E5 is 23,580 Da), when compared with a protein mixture used as standard (THY, thyroglobulin; FER, ferritin; ALD, aldolase; CON, conalbumin; OVA, ovalbumin; CAR, carbonic anhydrase; and RIB, ribonuclease). To calculate the molecular weight of E5, high and low molecular weight standard proteins were run in separate runs, and their molecular mass was plotted against the elution volume. The plot was fitted using an exponential function in Excel.

During their catalytic cycle, thioredoxins typically form an intramolecular disulfide bond in their hallmark CXXC motif (27). In our *in vitro* characterization experiments, one metagenomic thioredoxin representative did not show this expected reaction mechanism of thioredoxin superfamily members. Instead, it formed an intermolecular disulfide bond, resulting in dimer formation, which we could observe in our gel shift assays when we determined the redox potential of proteins. This particular thioredoxin, E5, which rescued the oxidase and isomerase phenotypes in *E. coli*, can also effectively replace the disulfide isomerase in a commercial strain used to fold challenging proteins (*E. coli* SHUFFLE). We tested the biotechnological potential of E5 in this strain with alkaline phosphatase (PhoA) and acidic phosphatase (AppA) from *E. coli*, urokinase, and a truncated version of human tissue plasminogen activator (vtPA) as substrate proteins. While E5 was as effective as DsbC to help form the correct disulfide bonds in PhoA and vtPA, it was significantly more effective in folding AppA (Fig. 8*A*).

*In vitro*, E5 showed the highest insulin reduction activity of all thioredoxins tested (Figs. 7*B* and 8*B*) and, most unusually, formed a dimer upon oxidation. We observed this dimer formation in nonreducing SDS-PAGE (Fig. 8, *C* and *D*).

To elucidate if the observed dimer was an artifact of our experimental setup, we asked, if it was also part of the catalytic cycle of E5. We thus used reduced E5 in a reaction with insulin, where the reductant DTT was ommitted (Fig. 8*E*). Under these conditions, E5 did, in fact, reduce insulin and formed a disulfide-linked dimer as well (Fig. 8*F*). We could exclude dimer formation through air oxidation, as no dimer formation was observed in the assay when omitting insulin. We thus concluded that dimer formation was indeed part of the catalytic cycle.

**Figure 9:**
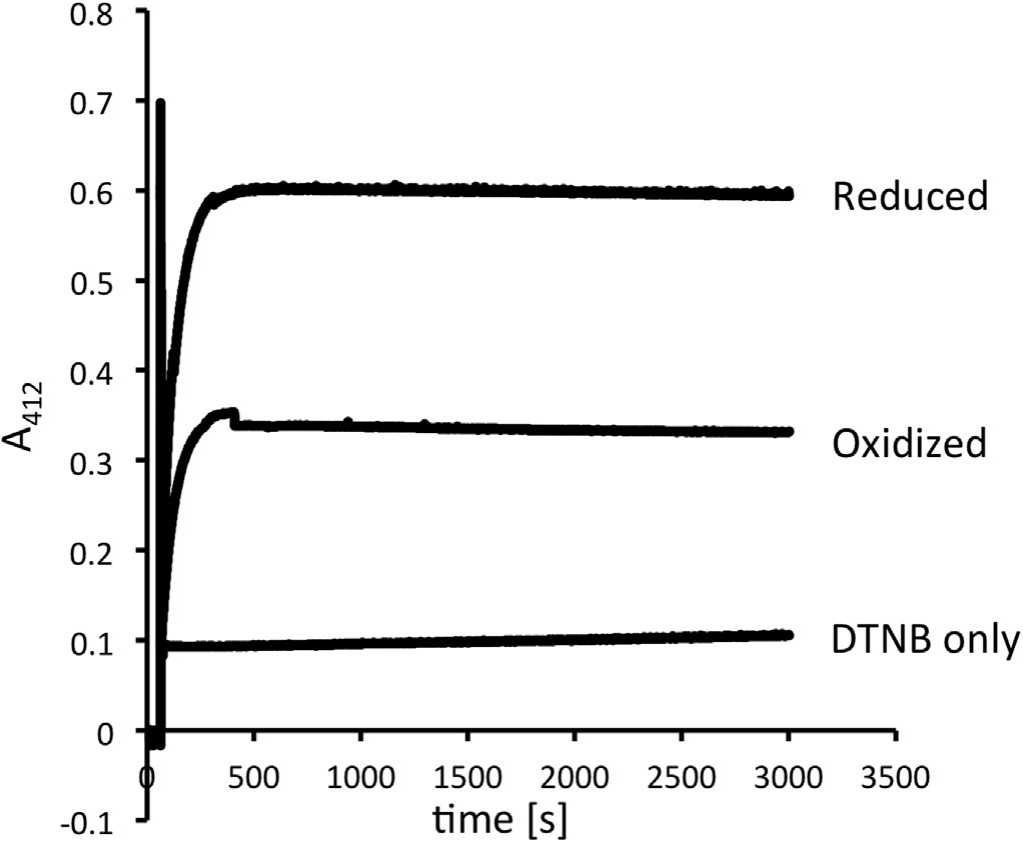
**Ellman's assay to determine free thiols in reduced and oxidized metagenomic thioredoxin representative E5.** DTNB and diamide-oxidized or DTT-reduced E5 protein (50 μM) were added to potassium phosphate buffer. As a control, no protein was added (DTNB only), and release of TNB was monitored at 412 nm over time. DTNB, dithionitrobenzoic acid.

Because E5 possesses two cysteines, both located in the active site, we argued that dimer formation must involve at least one cysteine from each subunit or even both cysteines. To test for these possibilities, we determined the presence of free cysteines in the reduced and oxidized forms of E5 in an Ellman's assay. This assay, which uses the propensity of free thiols to release the yellow compound thiol-nitro benzene from Ellman's reagent (5,5'-dithiobis(2-nitrobenzoic acid)), showed that the oxidized form contains half as many available cysteines per subunit than the reduced form, suggesting to us that only one cysteine from each subunit was involved in dimer formation (Fig. 9).

**Figure 10:**
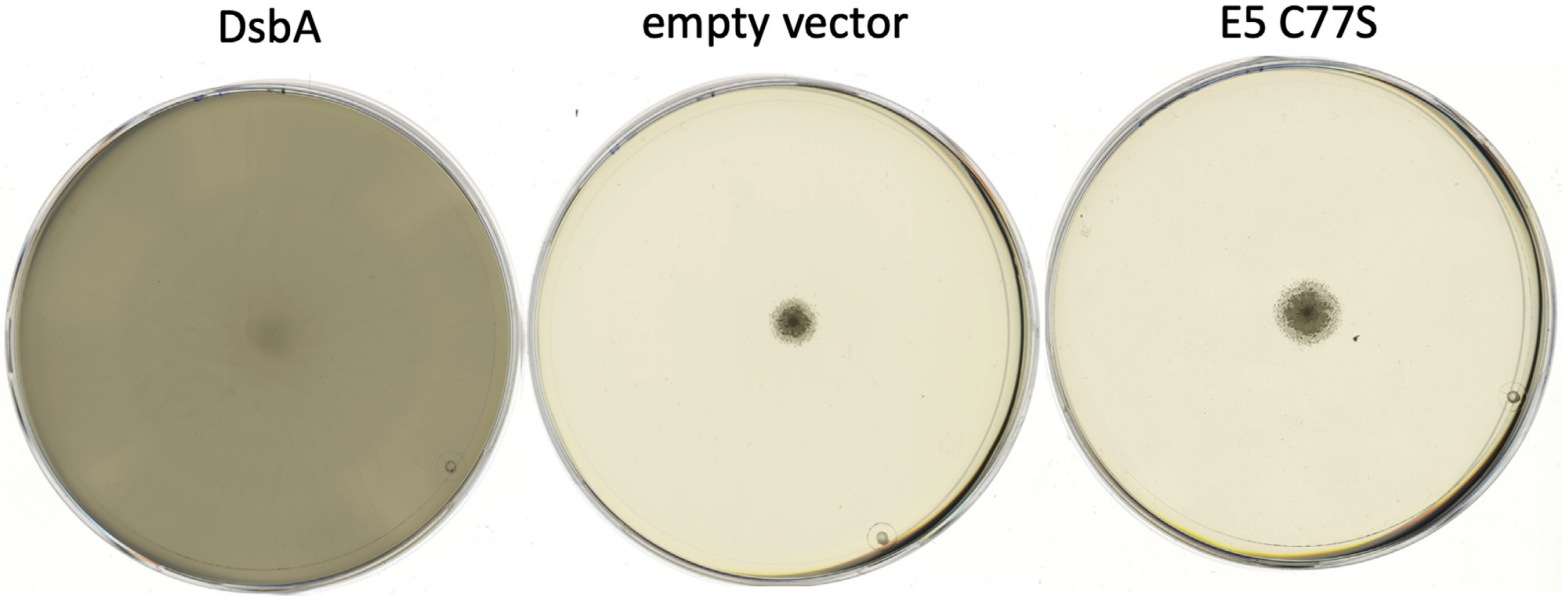
**An E5 C77S mutant lacking the resolving cysteine does not complement the DsbA phenotype**.

In thioredoxins, the N-terminal cysteine of the CXXC motif is considered the attacking cysteine and typically interacts with the substrate first, whereas the C-terminal cysteine is the resolving cysteine, completing the reaction cycle (28). We argued that instead of relying on the resolving cysteine, E5 could rely on the attacking cysteine of another E5 molecule to complete the reaction cycle. A mutant protein lacking the C-terminal resolving cysteine (C77S) should therefore still be able to form the disulfide-linked dimer upon oxidation. Although it does not rescue the DsbA phenotype in *E. coli* (Fig. 10), suggesting the resolving cysteine is needed *in vivo* for oxidase activity, it is indeed able to form the disulfide *in vitro* (Fig. 8*G*). More importantly, this mutant is still catalytically highly active in the insulin reduction assay (Fig. 8*H*). The presence of DTT in our insulin reduction assay could potentially compensate for the lack of the resolving cysteine under these circumstances, although the formation of a disulfide bond in the wildtype in the assay without DTT (Fig. 8*E*) indicates that DTT is not needed to resolve the insulin–E5 adduct. This is in contrast to typical thioredoxins, where mutants in the resolving cysteine of thioredoxins cannot complete their catalytic cycle, even in the presence of DTT. This fact has successfully been exploited to trap thioredoxin protein–substrate complexes for the identification of substrate proteins (29). In contrast, a C74S variant of E5 (lacking the attacking cysteine) did not show any activity in an insulin reduction assay (Fig. 8*H*).

To elucidate if E5 already forms a dimer under reducing conditions, we performed a size exclusion chromatography in the presence of a reducing agent. An E5 monomer has a molecular weight of 23,580 Da, and a BLAST search reveals a DsbA/Com1-like domain. DsbA itself is known to form a dimer. The elution profile of E5 and its C77S variant showed a peak at an apparent molecular weight of ∼153 KDa, consistent with a hexameric quaternary structure (Fig. 8*I*), suggesting, together with the observation of a covalent linkage upon oxidation, that E5 forms a trimer of dimers, each with its active site cysteines in close vicinity. Such a hexameric structure could enhance the propensity of intermolecular disulfide bond formation between the subunits. Given that DsbC is a natural dimer, the multimeric structure of E5 and its CGYC active site hint at a potential isomerase function in its unknown native host, with a redox potential that makes it prone to oxidation in the periplasm of *E. coli*.

## Conclusion

Our functional metagenomics approach applied to the GOS data set highlights the functional plasticity of the ancient thioredoxin superfamily by screening a select set of functions typical for these proteins. Depending on the cellular context, the *in vivo* function of a thioredoxin protein can change and even be reversed. The determined redox potential as well as insulin reduction activity do not correlate with *in vivo* activity but seems to be dependent on the protein's isoelectric surface potential. Our comprehensive screening revealed a novel thioredoxin-like protein. This protein only needs a single cysteine to function and relies on dimerization to complete its reaction cycle, has a hexameric quaternary structure, and shows higher activity in an insulin reduction assay than *E. coli* thioredoxin.

## Experimental procedures

### Definition of thioredoxin superfamily members from the GOS data set

All predicted protein sequences from the GOS data set were obtained from National Center for Biotechnology Information BioProject 13694 (accession no.: PRJNA13694). Redundancy of this data set was then reduced to 90% using Cd-Hit (30). The sequences from this nonredundant GOS data set were then matched to hidden Markov models of the Pfam thioredoxin clan CL0172 using HMMER (version 3.0) (11, 31). All sequences that matched with a score below 10^−5^ were considered for further analysis. Sequences that did not contain an unambiguous start or stop codon in their associated DNA scaffolds were removed to obtain only complete protein sequences. Sequences not containing at least one cysteine in the hallmark CXXC motif were removed, leaving a total of 4673 sequences. This set was then divided into 49 subfamilies using the Markov cluster clustering algorithm with an inflation parameter of I = 6 as described (12) (Table S1).

### Selection of representative sequences

Unweighted pair group method with arithmetic mean trees of each cluster were created using MAFFT (version 7) and saved in the Newick format (32). In these trees, the sum of the phylogenetic distance for each individual protein to all other proteins was calculated from the individual branch lengths in the tree file (see Supporting Information S5 for the lisp program used). The sequence with the smallest overall distance was chosen as the representative sequence. For clusters with more than 50 members, additional sequences were chosen, so that at least 50 and at most 100 sequences were associated with a representative sequence. Said additional sequences were chosen in an equidistantly distributed manner based on BLASTP bit scores between the representatives and all other cluster members (Table S1). About 96 protein sequences were denominated arbitrarily A1 to A12, B1 to B12, … , H1 to H12 based on positions in a microwell plate (Table S1). Four additional protein sequences were chosen by an intial BLASTP similarity search to *E. coli* TrxA (R1 and R2), DsbA (O1), and DsbC (I1) (National Center for Biotechnology Information GenInfo Identifier accession: 140401181, 143027041, 142228890, and 136774375). All protein sequences of synthesized genes can be found in Supporting Information S1, and accession numbers for all selected superfamily members found in the GOS data set can be found in Table S1.

### Codon optimization, sequence analyses, and gene synthesis

Codon usage was optimized for protein expression in *E. coli* with the help of the online tool JCat (33). Interfering restriction sites (*Nde*I, *Eco*RI, and *Bam*HI) were excluded during codon optimization. Incorrectly annotated N termini from mistakenly assigned start methionines in the metagenome-derived contigs were removed based on a comparison with homologous proteins using BLAST (34). If the start methionine of a substantial number of homologous proteins mapped to an internal methionine of the target gene, the N terminus was shortened accordingly prior to codon optimization. Leucine, isoleucine, or valine was taken into consideration as potential starting positions, since codons of these amino acids can function as alternative start codons (35). Prediction of potential signal sequences was done with the help of SignalP (36). Default parameters were used, except “Organism group,” which was set to “Gram-negative bacteria.” Sequence analysis (extinction coefficient calculation and molecular weight determination) was performed with the ProtParam tool (37). Gene synthesis was performed by Life Technologies (GeneArt).

### Strain construction and strain maintenance

*E. coli* strains and plasmids used in this study are listed in Table S3. For liquid cultures, an appropriate volume of LB medium (10 g/l tryptone, 10 g/l NaCl, and 5 g/l yeast extract) was inoculated with a single colony of the strain of interest and grown overnight at 37 °C, shaking at 125 rpm. Media were supplemented with 200 μg/ml kanamycin and/or 100 μg/ml ampicillin, if appropriate. This preculture was used to inoculate a main culture to an absorbance of 0.05 at 600 nm, and growth was followed spectrophotometrically. For long-term storage of strains, samples of overnight cultures were supplemented to 12% (v/v) glycerol and stored at −70 °C. For recultivation, glycerol stocks were streaked out on LB agar plates (LB medium with 15 g/l agar) containing the appropriate antibiotics and incubated overnight at 37 °C.

### Molecular cloning

In general, cloning procedures were carried out according to standard protocols (38). All enzymes for cloning work were obtained from Thermo Scientific. Genes were amplified *via* PCR generating necessary restriction sites through primer overhangs (primers and oligonucleotides used in this study are listed in Table S3). Genomic DNA of *E. coli* BW25113 served as template DNA for the amplification of *E. coli* genes (39). For the amplification of metagenes, standard vectors containing these synthesized metagenes were used as templates. For analysis and purification, PCR products were loaded on 1% agarose gels and separated by electrophoresis. PCR products were cut from the gel and purified using the NucleoSpin Extract II Kit (Macherey-Nagel). PCR product and target vector were digested with FastDigest enzymes according to the manufacturer's recommendations and purified on a gel as mentioned. Vector DNA was dephosphorylated with FastAP phosphatase, and insert DNA was phosphorylated with T4 polynucleotide kinase (PNK) according to the manufacturer's protocol and purified on a gel. Ligation was performed in 20 μl samples using T4 ligase according to the manufacturer's recommendations. Subsequently, competent *E. coli* XL1-blue cells were directly transformed with 4 μl of the ligation sample and finally streaked out on LB agar plates containing ampicillin. Clones were grown overnight at 37 °C, and plasmid constructs were isolated using the NucleoSpin Plasmid Kit by MACHEREY-NAGEL. Construct sequences were verified by DNA sequencing (Eurofins MWG Operon).

### Plasmids

Complementation vectors pCC and pPC were constructed by modifying the commercially available pTAC-MAT-Tag-2 vector (Sigma–Aldrich). For pCC, an *Nde*I restriction site was introduced by site-directed mutagenesis so that it contained the original start codon of the vector. The primers QCNde_For_neu and QCNde_Rev_neu were used (see Table S3 for primers used). Plasmid isolation and verification were performed as described previously. For pPC, the signal sequence of *E. coli ompA* was inserted between *Nde*I and *Hind*III sites of pCC according to the strategy described by Rentier-Delrue *et al.* (40). The *ompA* signal sequence (*ompA*ss, nucleotides 1–63) was amplified *via* PCR with primers ompASS_for(Nde) and ompASS(Hin)_rev. The *ompA*ss flanked by *Nde*I and *Hind*III sites was ligated into pCC. Plasmid isolation and verification were performed as described previously. In order to allow for standardized cloning *via Nde*I and *Eco*RI, an *Nde*I restriction site had to be introduced at the 3' end of the *ompA*ss. First, the *Nde*I site at the 5' was destroyed by site-directed mutagenesis with mutagenic primers pPCQCforward and pPCQCreverse. For the introduction of a new *Nde*I site at the 3' end of *ompA*ss, a round-the-horn mutagenesis was performed (41). First, primers pJH1-RTH-Forward and pJH1-RTH-Reverse were phosphorylated by mixing for each primer 1× PNK buffer, 1 mM MgSO_4_, 10 μM of primer, 2 mM ATP, and 10 U PNK. After incubation for 30 min at 37 °C, PNK was heat inactivated for 10 min at 75 °C. Then, a PCR with 1× Pfu Turbo polymerase, 0.3 μM of each primer, 200 μM deoxynucleotide triphosphates, 100 ng pPC (after site-directed mutagenesis from previously shown), and 2.5 U Pfu Turbo polymerase were performed using the following program: 98 °C/1 min (96 °C/30 s, 55 °C/30 s, and 68 °C/10 min for 30 s) for 25 cycles. Plasmid isolation and verification were performed as described previously. Commercially available pET11a (Novagen) was used to design pOE. First, the single *Eco*RI site within pET11a was destroyed using site-directed mutagenesis and mutagenic primers pETdestroyEco_f and pETdestroyEco_r. Then, an *Eco*RI site was introduced between the single *Nde*I site and the T7 terminator using the mutagenic primers pETintrodEco_f and pETintrodEco_r. Finally, a double-stranded oligonucleotide, which contained a His_6_ tag and a TEV protease cleavage site (ENLYFQG), was introduced between *Nde*I and *Xba*I. Two complementary oligonucleotides that contained *Nde*I and *Xba*I overhangs were mixed in equimolar amounts, heated to 95 °C for 2 min, and cooled on ice for 2 min. Subsequently, the insert was ligated into linearized vector as described previously. Plasmid isolation and verification was performed as described previously.

### Reductase assay

Reductase activity was tested in liquid M9 minimal medium containing 5 μM IPTG for the induction of protein expression. M9 minimal medium was prepared (38). Single colonies of *E. coli* Δ*trxA* Δ*gor* strains carrying different plasmid constructs were transferred to the medium and incubated for 2 days at 30 °C in a roller drum. Growth was determined sprectrophotometrically at 600 nm. These assays were performed in three independent experiments. Strains that reached overnight an absorbance of > 0.2 at 600 nm in all experiments were considered to express a metagenomic reductase.

### Oxidase assay

For the oxidase activity assay, semisolid LB agar plates (0.35% [w/v] agar) containing 5 μM IPTG were prepared. Single colonies of *E. coli* Δ*dsbA* mutant strains carrying different plasmid constructs were transferred to the center of the plates. Then, plates were incubated for 24 h at 37 °C. After incubation, plates were inspected for cell halos indicating DsbA substitution. Strains that produced halos around the inoculation site in three independent experiments were considered to express a metagenomic oxidase.

### Isomerase assay

For the isomerase activity assay, Mops agar plates containing 50 μM IPTG were prepared. Mops medium was obtained from Teknova and prepared according to Neidhardt *et al.* (42). Single colonies of *E. coli* Δ*dsbC* Δ*mdoG* double-mutant strains carrying different plasmid constructs were streaked out on the plates followed by an incubation for 24 h at 30 °C. Three independent experiments were performed, and only strains that showed a mucoid phenotype in all three experiments were considered to express a metagenomic isomerase.

### Secondary structure prediction and isosurface calculation

The secondary structure of thioredoxins was predicted using SWISS-MODEL (43). For isosurface calculation, the sequences of the 103 selected thioredoxin superfamily members were blinded, and the secondary structure was predicted using the automodel function of SWISS-MODEL (43). Computation of the protein electrostatic properties from the protein data bank (PDB) files were done as follows: the reconstruction of any missing atoms, the addition of hydrogens, and the assignment of atomic charges and radii were performed using pdb2pqr with the amber force field (44). All resulting secondary structures were aligned and oriented in the visual molecular dynamics program VMD (45) based on the structures of *E. coli* TrxA (PDB entry 1xoa) or *E. coli* Grx1 (PDB entry 1egr). Within the VMD software package, the Adaptive Poisson–Boltzmann Solver (46) was used to calculate the electrostatic potential that is visualized as isosurface and also mapped to the protein surface. Parameters for the calculations were set to 298 K and 150 mM mobile ions in aqueous solution. Images depicting the secondary structure (N-terminal active site cysteinyl residue facing toward camera perspective), the electrostatic potential mapped to the surface (from −4 KT·e^−1^ in *red* to 4 KT·e^−1^ in *blue*), and the isosurface of the electrostatic potential (from −1 KT·e^−1^ in *red* to 1 KT·e^−1^ in *blue*) were rendered.

The hydrophobicity of the surface was analyzed using Chimera (47). The computation was done using the amino acid hydrophobicity in the Kyte–Doolittle scale depicted in *red* (hydrophobic) and *blue* (hydrophilic). An overview of all blinded structures (Fig. S1) was independently evaluated by two researchers to predict functional candidates. Using this strategy, unblinding revealed that 11 of 16 structures of proteins that functioned in the Δ*gor*/Δ*trxA* complementation assay were predicted correctly (Fig. S1, marked with an *asterisk*) with no false positives. No isosurfaces could be calculated for A2, A9, B8, E10, and F6, as no suitable model for structure prediction was found.

### Protein purification

The genes of proteins, which were predicted to possess a signal sequence, were cloned into pOE with the signal sequence removed. This was the case for O1, I1, H3, H8, B11, D6, D11, and E5. Single colonies of *E. coli* BL21(DE3) carrying the respective overexpression plasmids were inoculated in 50 ml LB medium containing ampicillin and grown overnight at 37 °C. Then, a 5 l main culture was inoculated with the preculture and incubated to an absorbance of 0.5 at 600 nm at 37 °C. About 1 mM of IPTG and fresh ampicillin were added, and the culture was incubated overnight at 20 °C. Cells were harvested by centrifugation (30 min at 4 °C, 7800*g*). The cell pellet was resuspended in 100 ml of buffer A (50 mM sodium phosphate, 300 mM NaCl, pH 8.0) including two dissolved pills of an EDTA-free protease inhibition cocktail (Roche) and 1 mM PMSF. Then, cells were disrupted using a high-pressure cell disruption system at 1.9 kbar (Constant Systems). Cell lysate was centrifuged for 1 h at 4 °C and 67,500*g*. Supernatant was filtered using “Filtropur” 500 ml vacuum filters (Sarstedt). Purification and fractionation steps were performed with an “ÄKTApurifier” FPLC system (GE Healthcare). Filtrate was loaded onto 2 × 5 ml nickel–nitrilotriacetic acid (Ni–NTA) columns “HisTrapTM HP” (GE Healthcare) equilibrated with buffer A. Elution was performed with six column volumes of buffer B (buffer A containing 500 mM imidazole). About 3-ml fractions were collected and analyzed by SDS-PAGE. Fractions with the highest target protein yield were pooled, and His_6_-tagged TEV protease was added (30-fold excess of target protein to TEV protease). Protein solution was dialyzed against buffer A in Spectra/Por dialysis tubes with a pore size of 6 to 8000 molecular weight cutoff (spectrumlabs.com; Breda, The Netherlands), overnight at 4 °C. Then, the protein solution was loaded onto the Ni–NTA columns to remove the His_6_-tagged TEV protease as well as the cleaved His_6_ tag. Flowthrough containing the protein was dialyzed against 100 mM sodium phosphate buffer (pH 7.0) containing 1 mM EDTA and concentrated in Amicon Ultra concentrators pore size 3000 molecular weight cutoff (Millipore).

Overexpression and purification was modified for R1, since it was highly insoluble in test expression experiments. A single colony of *E. coli* BL21(DE3) carrying pSN61 was transferred into 5 ml LB medium containing ampicillin and incubated for 90 min at 37 °C. Then, cell suspension was transferred into a 5 l main culture and grown overnight at 37 °C without shaking. Then, the culture was grown under shaking to an absorbance of 0.5 at 600 nm, and IPTG induction was performed as described previously. After growth for 4 h at 37 °C, cells were harvested as described previously. The cell pellet was resuspended in buffer A. Cell disruption and centrifugation were performed as described previously. The insoluble pellet was resuspended in 50 ml of 8 M urea. This protein solution was slowly dissolved in 5 l PBS (pH 7.4) and stirred at 4 °C overnight for refolding. Then, protein solution was centrifuged for 1 h at 4 °C and 15,500*g* to remove remaining unfolded protein. The supernatant was filtered as described previously. The entire filtrate was loaded onto one 5 ml Ni–NTA column overnight at 4 °C using a peristaltic pump. Elution and further purification steps were performed as described previously. Buffer A was supplemented with 2 mM β-mercaptoethanol.

Proteins H3, B11, H8, D6, D11, and E5 were predicted to contain a signal sequence. For cytoplasmic overexpression, predicted signal sequences were removed by PCR and genes cloned into pOE as described previously. Overexpression was done as for O1, I1, and R1 but on a 1 L scale. Cleared cell lysate was loaded onto a self-packed column using 1 ml Ni–NTA agarose (Qiagen) in Poly-Prep Chromatography Columns (Bio-Rad). Purification was performed according to the manufacturer's protocol. This protocol was also used for C4, C12, B1, A8, H11, E1, D4, F12, G5, and the C-terminal cysteine mutant of E5. After purification, His_6_ tag removal was achieved as described previously. However, His_6_ tag removal could not be successfully achieved for B1, C4, D6, D11, E5, and G5, presumably because of structural inaccessibility. These proteins were characterized still containing the His_6_ tag.

### Insulin reduction assay

FITC-labeled insulin reduction was monitored fluorometrically with a fluorescence spectrophotometer FP-8500 (Jasco) equipped with a Peltier-thermo cell holder EHC-813. Emission was followed over time at a wavelength of 520 nm. Excitation wavelength was 490 nm. Emission and excitation bandwidth was 2.5 nm, response was 1 s, and sensitivity was low. Samples were prepared in a quartz cuvette Suprasil for fluorescence measurements (Hellma). General sample composition was adopted from Holmgren (23) and slightly modified using FITC-labeled insulin (Sigma–Aldrich). Potassium phosphate buffer (pH 7.0) of 20 mM, 2 mM EDTA, 330 μM DTT, and 10 μg/ml FITC-labeled insulin were added in that order, and the measurement was immediately started at 25 °C while stirring (800 rpm). After the fluorescent signal was stable, the protein of interest was added to a final concentration of 1 μM. As control, protein was omitted to follow uncatalyzed reduction by DTT. For one reaction cycle catalysis, DTT was left out and reduced protein was added. Protein was reduced using 1 mM DTT, and DTT was subsequently removed using an “illustra NAP-5 column” (GE Healthcare).

The initial slope of fluorescence increase after addition of proteins was used to determine protein activity (rate of FITC-labeled insulin reduction per time per mol protein). The initial slopes were determined with the “spectra analysis/kinetics” mode of the measurement control and evaluation program Spectra Manager 2.09.01 (Jasco). Activity of *E. coli* TrxA was set to 100%.

### Redox potential determination

In an anerobic chamber (Coy Laboratories), proteins of interest (20 μM) were completely oxidized with 1 mM diamide for 1 h at 37 °C to produce fully oxidized protein. Then, diamide was removed using an “illustra NAP-5 column” (GE Healthcare). Proteins were eluted from the column in 100 mM sodium phosphate buffer (pH 7.0) containing 1 mM EDTA. Then, they were mixed with GSH/GSSG redox buffers containing increasing GSH concentrations (1 μM–200 mM) and constant GSSG concentrations (100 μM in case of O1 and 10 μM in all other cases). Control samples containing only GSH and only GSSG were prepared to fully reduce and fully oxidize proteins. The pH of samples containing GSH concentrations above 50 mM was adjusted to pH 7.0 by adding concentrated NaOH. Samples were incubated overnight at 25 °C under slight shaking at 400 rpm in a Thermo Mixer (Eppendorf). After incubation, proteins were precipitated with 10% (w/v) trichloroacetic acid (TCA) for 20 min on ice. Samples were spun down for 30 min at 4 °C and 18,000*g*. The supernatant was discarded, and the pellet was washed in 60 μl of 10% TCA and 20 μl of 5% TCA. In a final centrifugation step, the protein pellet was dried completely to remove any residual TCA. Protein pellets were then resuspended in 20 μl of 50 mM AMS in denaturing alkylation buffer (6 M urea, 200 mM Tris–HCl (pH 8.5), 10 mM EDTA, and 0.5% [w/v] SDS) (48). In a ThermoMixer, samples were incubated for 1 h at 25 °C and 1300 rpm in the dark. Then, samples were incubated for 5 min at 95 °C in 1× nonreducing SDS loading buffer (2% [w/v] SDS, 12% [v/v] glycerol, 60 mM Tris–HCl [pH 7.0], and 0.0001% [w/v] bromophenol blue) and loaded onto a precast NuPAGE Novex 4 to 12% Bis–Tris protein gel (Life Technologies). The gel was run under nonreducing conditions at 300 V and 110 mA and Coomassie stained.

Evaluation of protein band patterns was performed by integrated density measurements using Photoshop CS5 Extended 12.0 (Adobe) and ImageJ 1.46r (49). The running position of protein oxidation states was determined by the band patterns for fully oxidized and fully reduced proteins. The rectangle tool was used to fully enclose the protein bands. For each sample, bands for oxidized and reduced protein were selected, and optical parameters were measured automatically. The integrated density was used as readout for the protein amount. The differences between reduced and oxidized protein proportions were compared with values for fully oxidized or reduced samples. These samples were set to 100% oxidized or reduced, respectively. For redox equilibrium determination, values of reduced protein proportion (in percent) were logarithmically plotted against the concentration ratio of reduced and oxidized glutathione [GSH]^2^/[GSSG]. The average of the initial and final values was set to 0% and 100% reduced, respectively. These scaled curves were fitted using Igor Pro 6.32A (Wavemetrics), and the [GSH]^2^/[GSSG] ratio at the point of inflection of the curve was used for the standard redox potential determination. The Nernst equation was applied assuming a standard redox potential of the GSH/GSSG couple of −240 mV (50).

### Quikchange mutagenesis of E5

To exchange cysteines within the CXXC motif of E5 to serine, Quikchange mutagenesis was performed as described previously. Transformation and sequence verification were performed as described previously.

### Size exclusion chromatography

Analytical gel filtration was performed as previously described (51). Briefly, E5 and E5 C77S was separated on an ÄKTA FPLC system running a Superdex 75 10/300 GL column (GE Healthcare) at 0.5 ml min^−1^ in 50 mM phosphate buffer containing 150 mM NaCl.

## Data availability

All data, such as sequence data and accession numbers, are contained within the article and the supporting information.

## Conflict of interest

The authors declare that they have no conflicts of interest with the contents of this article.
