# Supplementary figures and images for: Functional metagenomics of the thioredoxin superfamily (part 1 of 2)
Source: J Biol Chem. 2021 Jan 14;296:100247. doi: 10.1074/jbc.RA120.016350 (PMC7949104; doi:10.1074/jbc.RA120.016350)

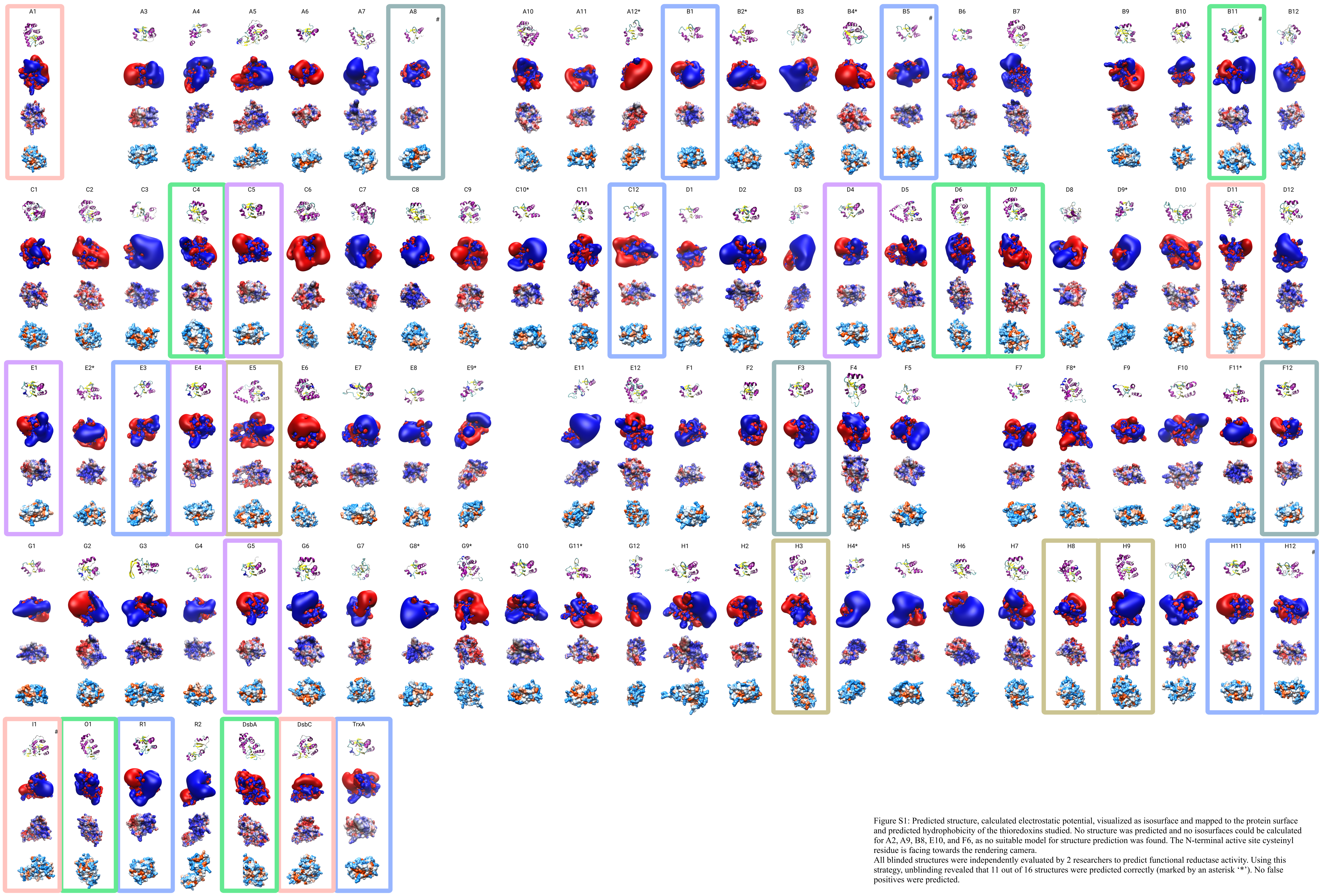

Supplement: Supplemental Figure S1 [file mmc1.pdf]

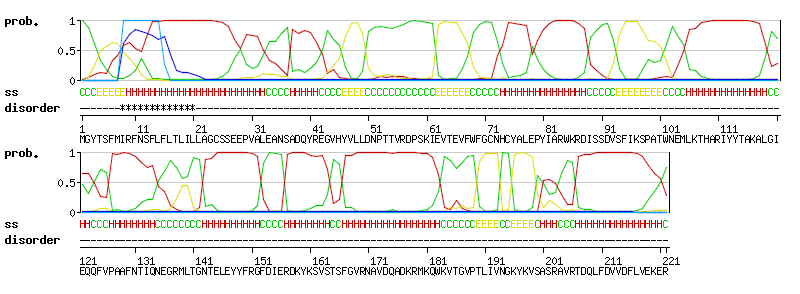

Supplement: Supporting Information S4 [file mmc6.zip › S4_Nilewski_et_al_Predicted_Secondary_Structure/D6.gif]

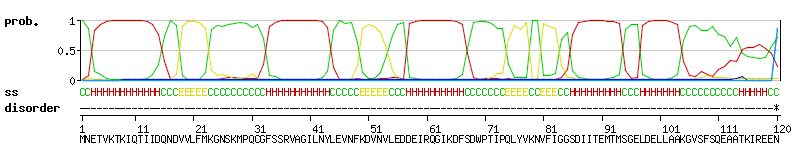

Supplement: Supporting Information S4 [file mmc6.zip › S4_Nilewski_et_al_Predicted_Secondary_Structure/E2.gif]

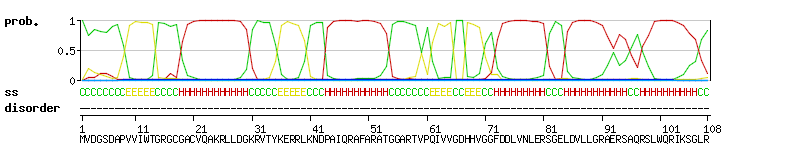

Supplement: Supporting Information S4 [file mmc6.zip › S4_Nilewski_et_al_Predicted_Secondary_Structure/H5.gif]

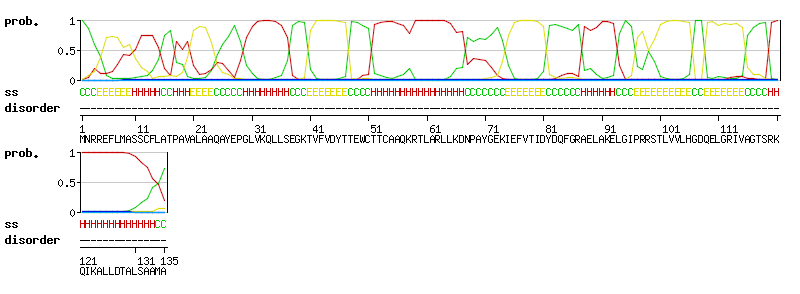

Supplement: Supporting Information S4 [file mmc6.zip › S4_Nilewski_et_al_Predicted_Secondary_Structure/B11.gif]

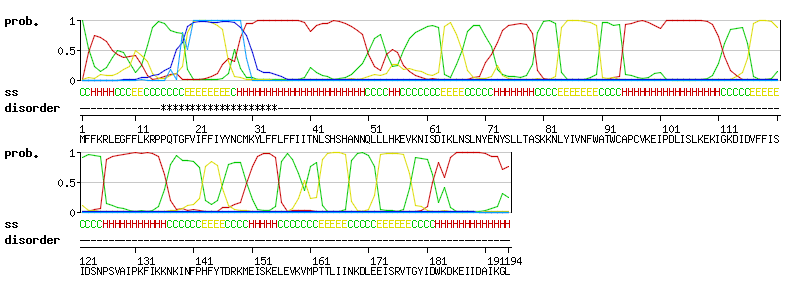

Supplement: Supporting Information S4 [file mmc6.zip › S4_Nilewski_et_al_Predicted_Secondary_Structure/B10.gif]

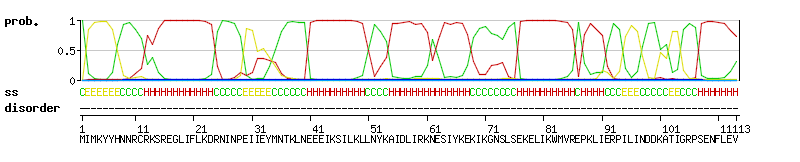

Supplement: Supporting Information S4 [file mmc6.zip › S4_Nilewski_et_al_Predicted_Secondary_Structure/H4.gif]

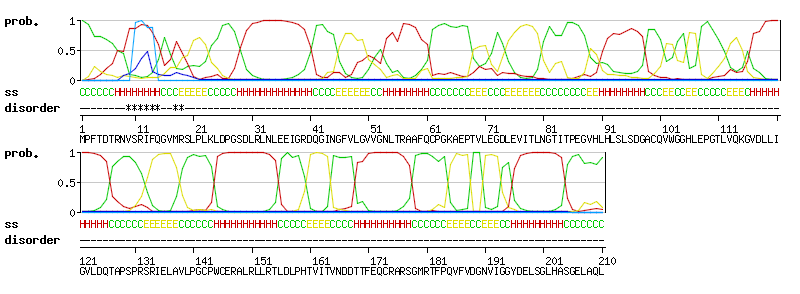

Supplement: Supporting Information S4 [file mmc6.zip › S4_Nilewski_et_al_Predicted_Secondary_Structure/E3.gif]

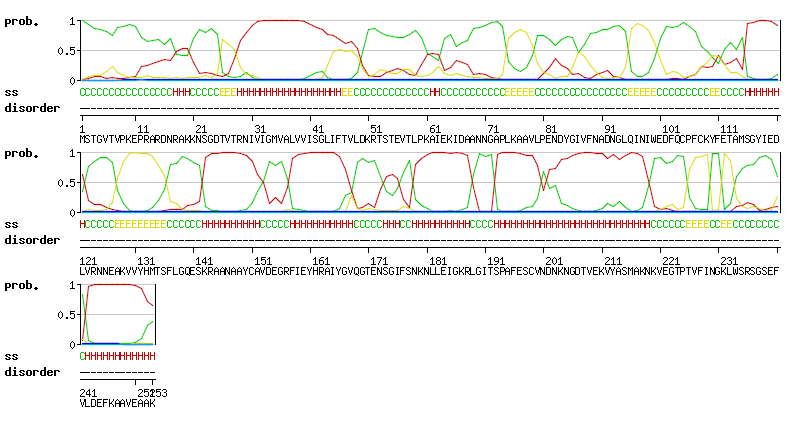

Supplement: Supporting Information S4 [file mmc6.zip › S4_Nilewski_et_al_Predicted_Secondary_Structure/D7.gif]

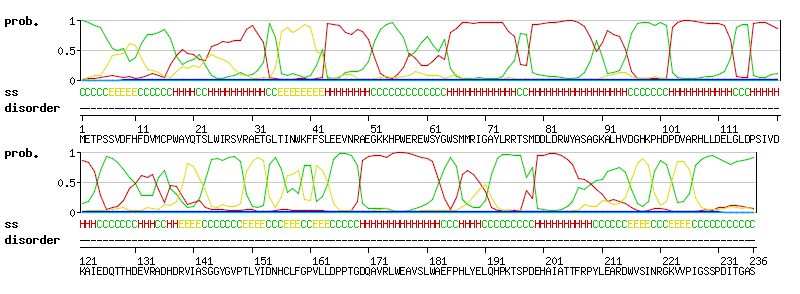

Supplement: Supporting Information S4 [file mmc6.zip › S4_Nilewski_et_al_Predicted_Secondary_Structure/G9.gif]

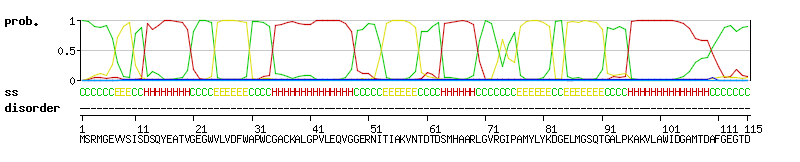

Supplement: Supporting Information S4 [file mmc6.zip › S4_Nilewski_et_al_Predicted_Secondary_Structure/E1.gif]

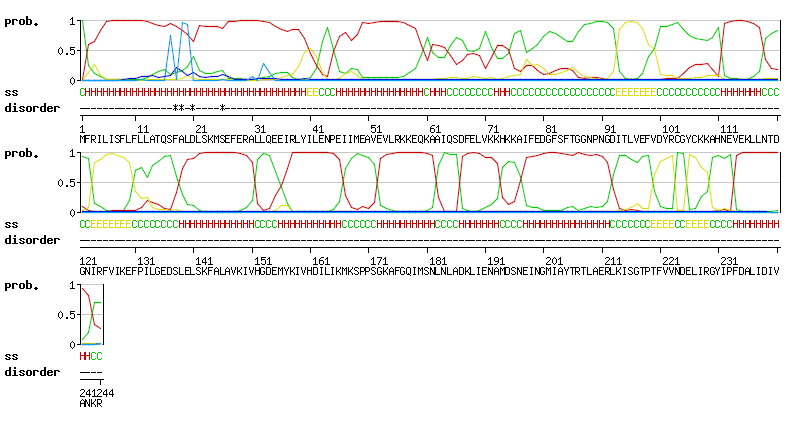

Supplement: Supporting Information S4 [file mmc6.zip › S4_Nilewski_et_al_Predicted_Secondary_Structure/D5.gif]

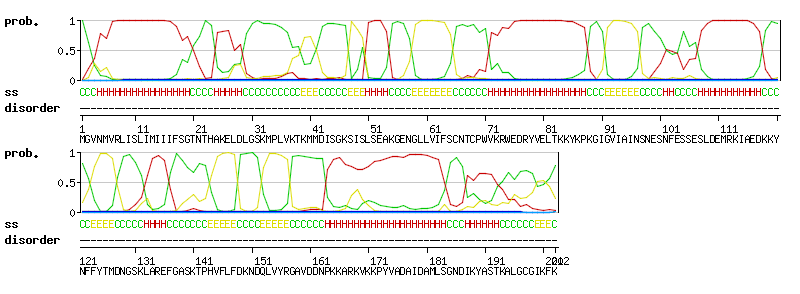

Supplement: Supporting Information S4 [file mmc6.zip › S4_Nilewski_et_al_Predicted_Secondary_Structure/H6.gif]

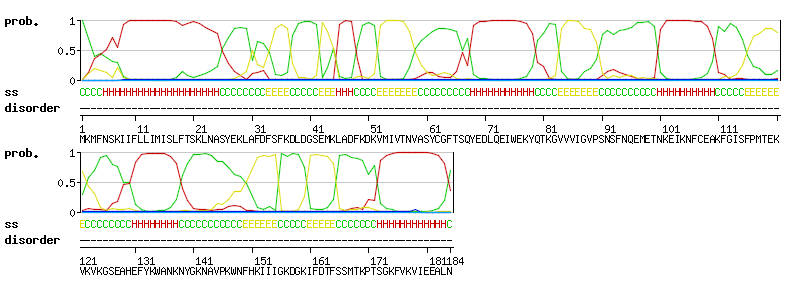

Supplement: Supporting Information S4 [file mmc6.zip › S4_Nilewski_et_al_Predicted_Secondary_Structure/B12.gif]

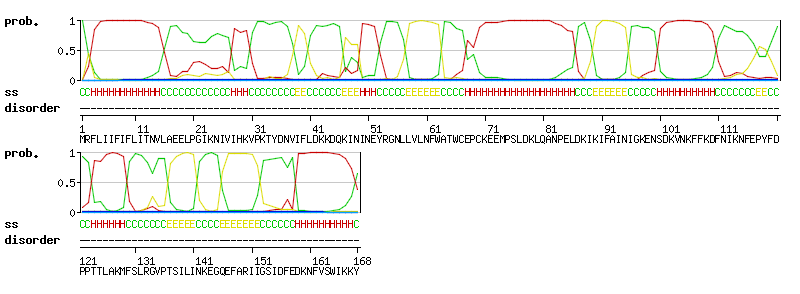

Supplement: Supporting Information S4 [file mmc6.zip › S4_Nilewski_et_al_Predicted_Secondary_Structure/H7.gif]

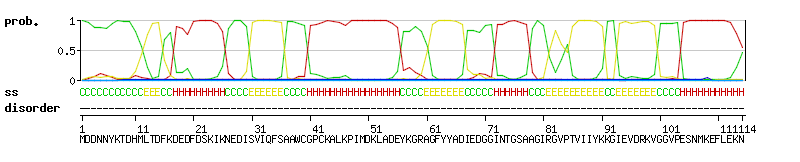

Supplement: Supporting Information S4 [file mmc6.zip › S4_Nilewski_et_al_Predicted_Secondary_Structure/D4.gif]

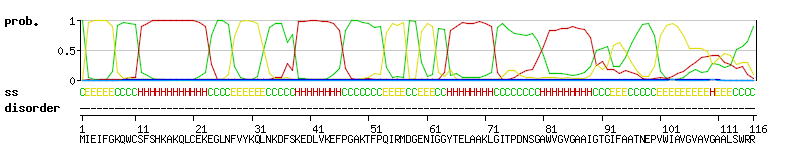

Supplement: Supporting Information S4 [file mmc6.zip › S4_Nilewski_et_al_Predicted_Secondary_Structure/G8.gif]

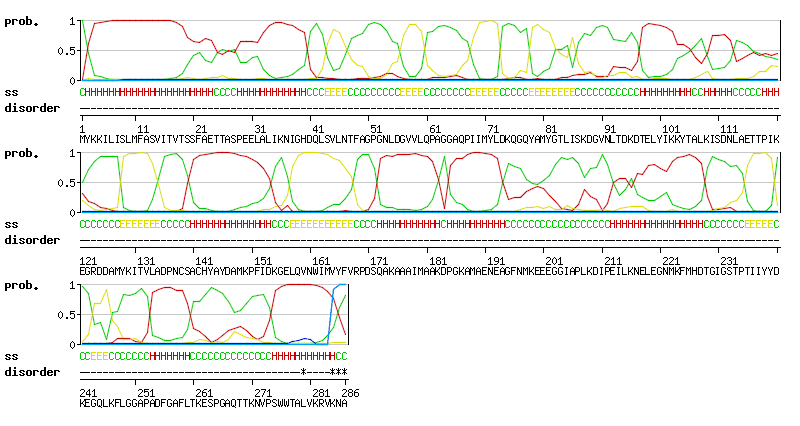

Supplement: Supporting Information S4 [file mmc6.zip › S4_Nilewski_et_al_Predicted_Secondary_Structure/D11.gif]

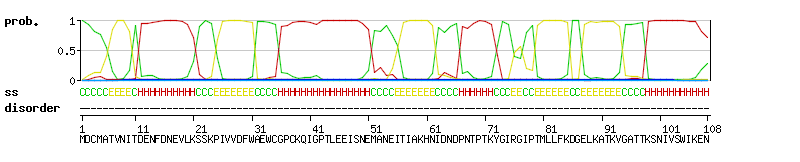

Supplement: Supporting Information S4 [file mmc6.zip › S4_Nilewski_et_al_Predicted_Secondary_Structure/E4.gif]

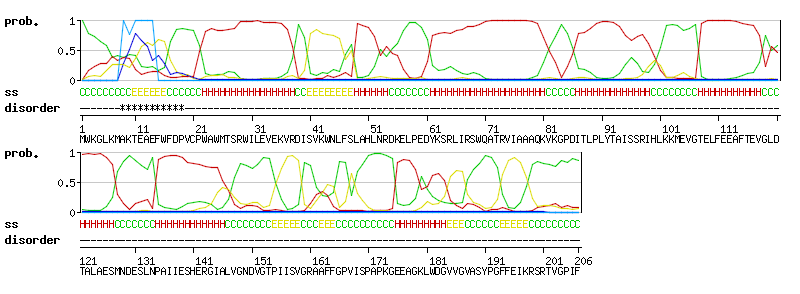

Supplement: Supporting Information S4 [file mmc6.zip › S4_Nilewski_et_al_Predicted_Secondary_Structure/F8.gif]

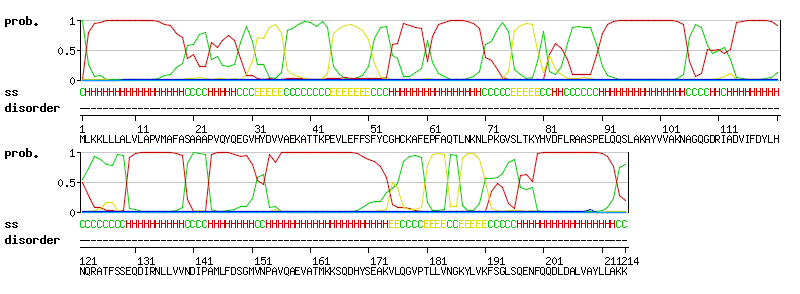

Supplement: Supporting Information S4 [file mmc6.zip › S4_Nilewski_et_al_Predicted_Secondary_Structure/H3.gif]

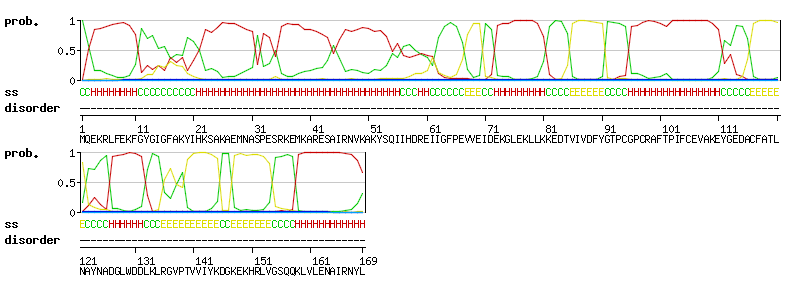

Supplement: Supporting Information S4 [file mmc6.zip › S4_Nilewski_et_al_Predicted_Secondary_Structure/H2.gif]

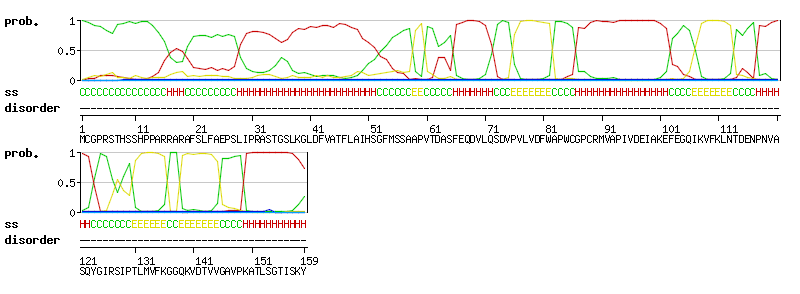

Supplement: Supporting Information S4 [file mmc6.zip › S4_Nilewski_et_al_Predicted_Secondary_Structure/F12.gif]

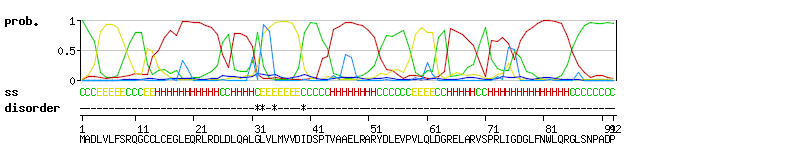

Supplement: Supporting Information S4 [file mmc6.zip › S4_Nilewski_et_al_Predicted_Secondary_Structure/F9.gif]

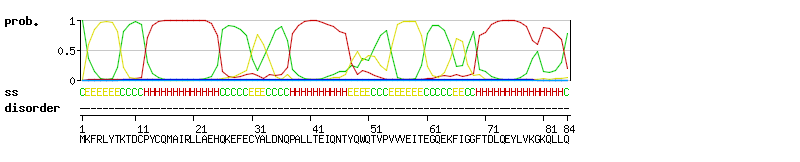

Supplement: Supporting Information S4 [file mmc6.zip › S4_Nilewski_et_al_Predicted_Secondary_Structure/D1.gif]

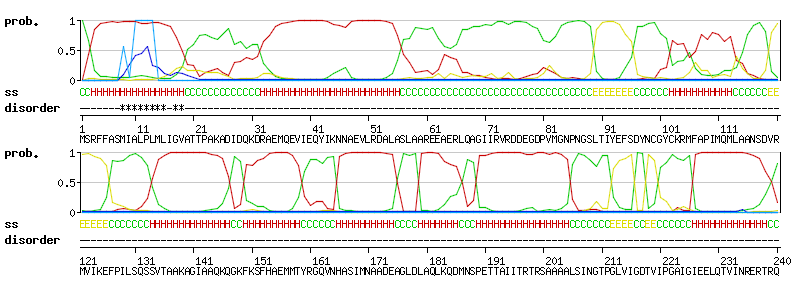

Supplement: Supporting Information S4 [file mmc6.zip › S4_Nilewski_et_al_Predicted_Secondary_Structure/E5.gif]

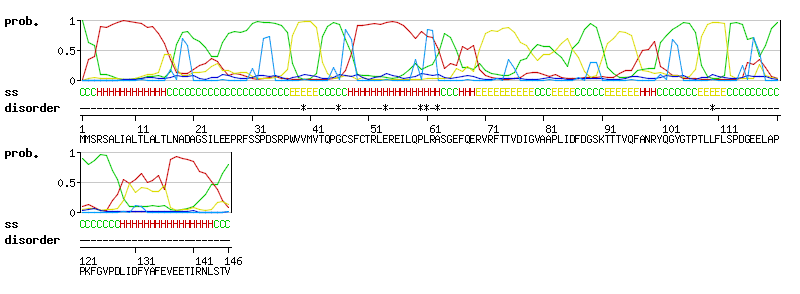

Supplement: Supporting Information S4 [file mmc6.zip › S4_Nilewski_et_al_Predicted_Secondary_Structure/D10.gif]

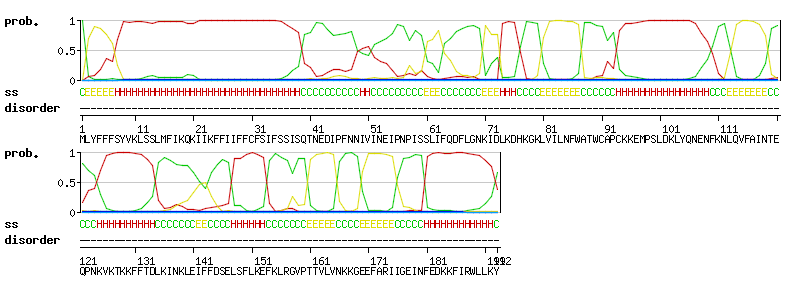

Supplement: Supporting Information S4 [file mmc6.zip › S4_Nilewski_et_al_Predicted_Secondary_Structure/D12.gif]

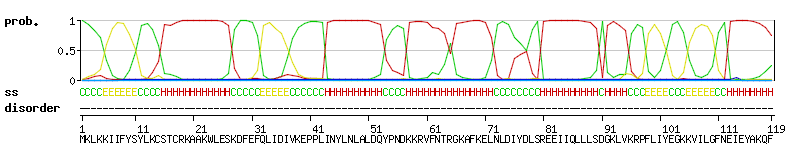

Supplement: Supporting Information S4 [file mmc6.zip › S4_Nilewski_et_al_Predicted_Secondary_Structure/D3.gif]

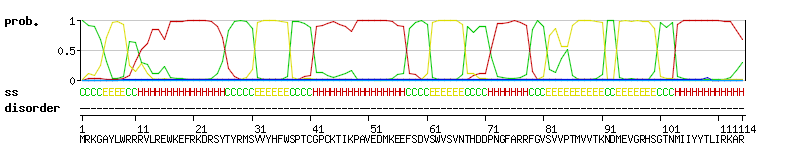

Supplement: Supporting Information S4 [file mmc6.zip › S4_Nilewski_et_al_Predicted_Secondary_Structure/E7.gif]

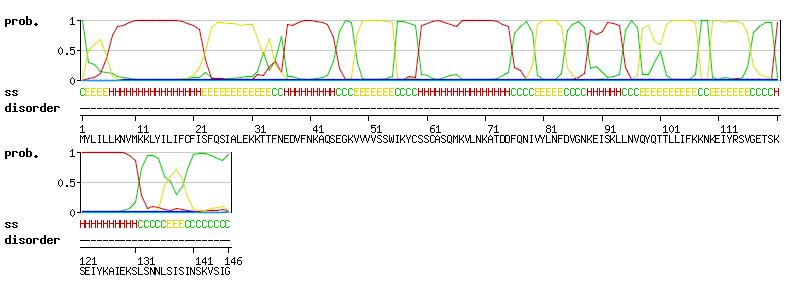

Supplement: Supporting Information S4 [file mmc6.zip › S4_Nilewski_et_al_Predicted_Secondary_Structure/F10.gif]

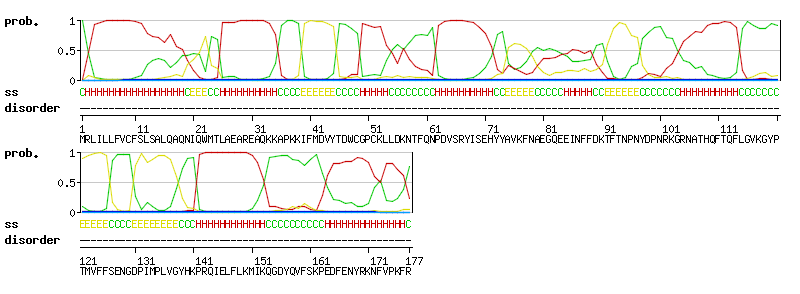

Supplement: Supporting Information S4 [file mmc6.zip › S4_Nilewski_et_al_Predicted_Secondary_Structure/H1.gif]

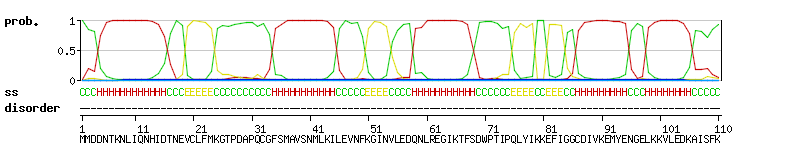

Supplement: Supporting Information S4 [file mmc6.zip › S4_Nilewski_et_al_Predicted_Secondary_Structure/F11.gif]

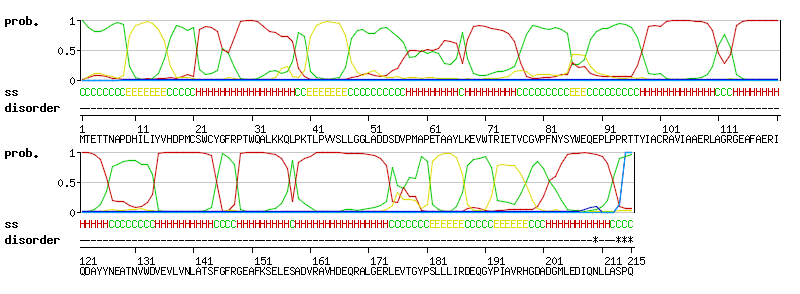

Supplement: Supporting Information S4 [file mmc6.zip › S4_Nilewski_et_al_Predicted_Secondary_Structure/E6.gif]

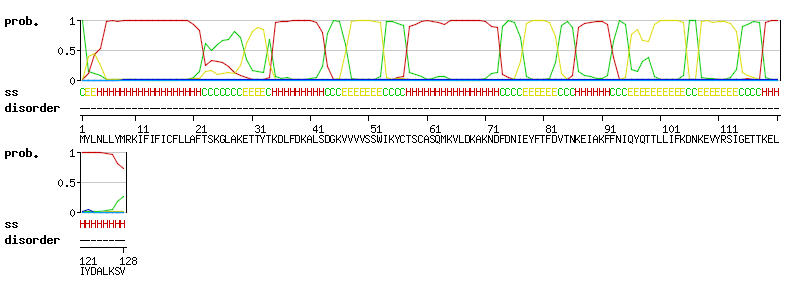

Supplement: Supporting Information S4 [file mmc6.zip › S4_Nilewski_et_al_Predicted_Secondary_Structure/D2.gif]

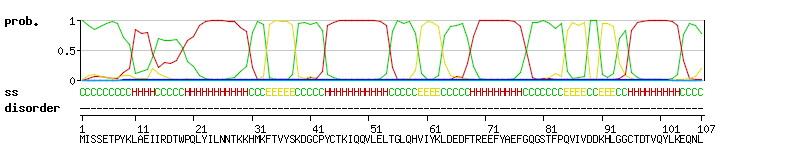

Supplement: Supporting Information S4 [file mmc6.zip › S4_Nilewski_et_al_Predicted_Secondary_Structure/C12.gif]

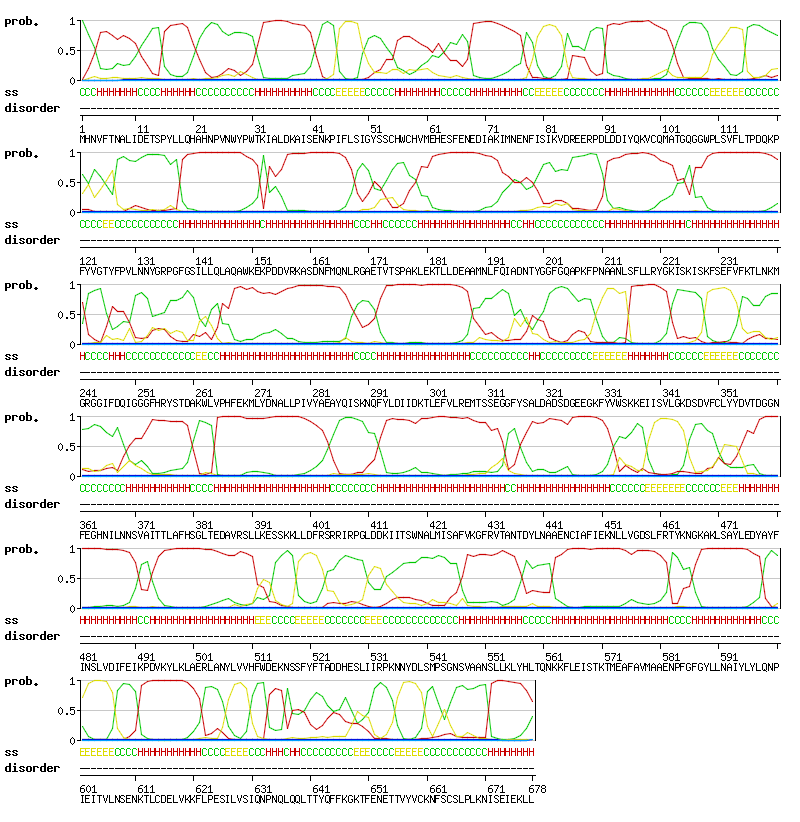

Supplement: Supporting Information S4 [file mmc6.zip › S4_Nilewski_et_al_Predicted_Secondary_Structure/A9.gif]

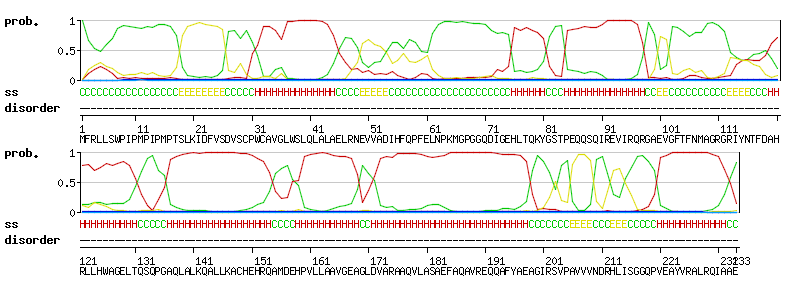

Supplement: Supporting Information S4 [file mmc6.zip › S4_Nilewski_et_al_Predicted_Secondary_Structure/A10.gif]

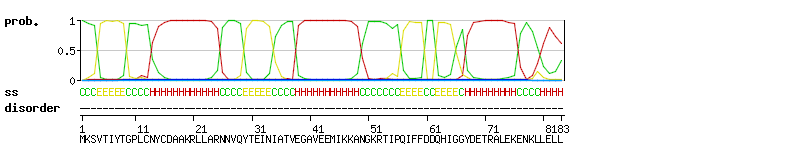

Supplement: Supporting Information S4 [file mmc6.zip › S4_Nilewski_et_al_Predicted_Secondary_Structure/B5.gif]

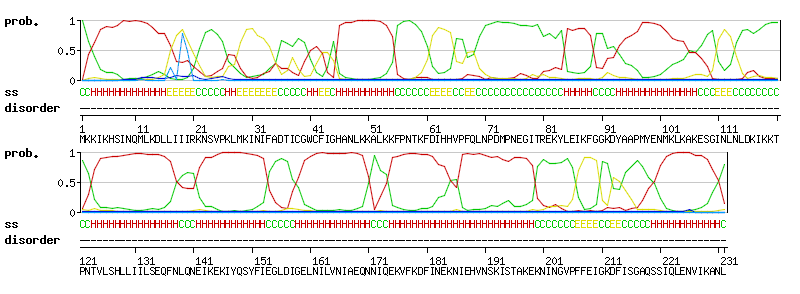

Supplement: Supporting Information S4 [file mmc6.zip › S4_Nilewski_et_al_Predicted_Secondary_Structure/C1.gif]

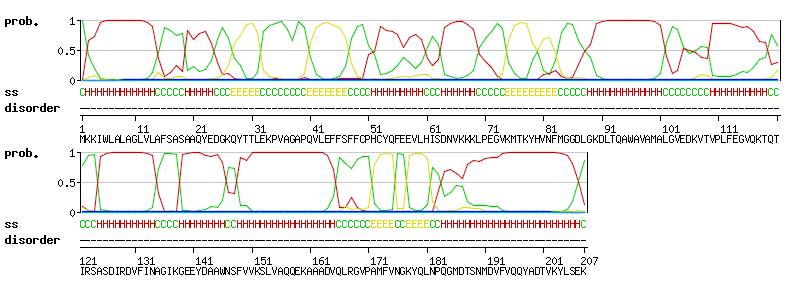

Supplement: Supporting Information S4 [file mmc6.zip › S4_Nilewski_et_al_Predicted_Secondary_Structure/DsbA.gif]

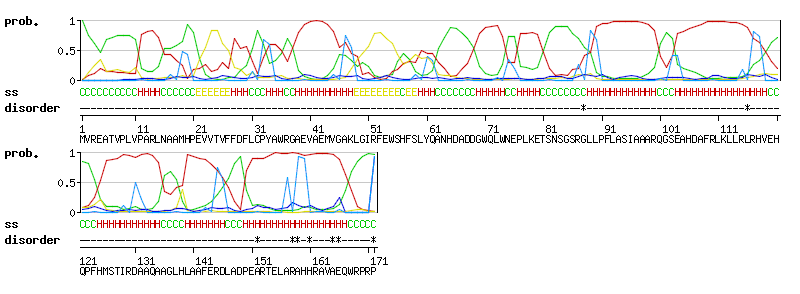

Supplement: Supporting Information S4 [file mmc6.zip › S4_Nilewski_et_al_Predicted_Secondary_Structure/B4.gif]

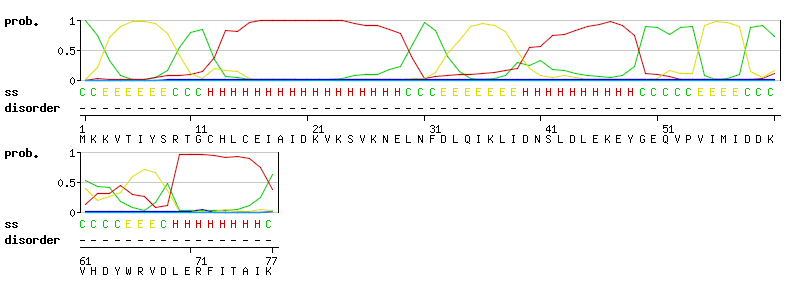

Supplement: Supporting Information S4 [file mmc6.zip › S4_Nilewski_et_al_Predicted_Secondary_Structure/A11.gif]

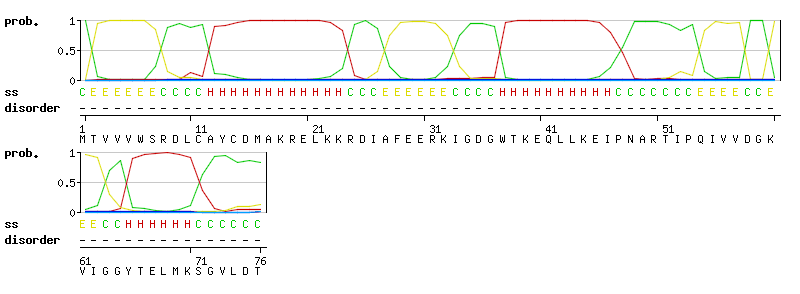

Supplement: Supporting Information S4 [file mmc6.zip › S4_Nilewski_et_al_Predicted_Secondary_Structure/A8.gif]

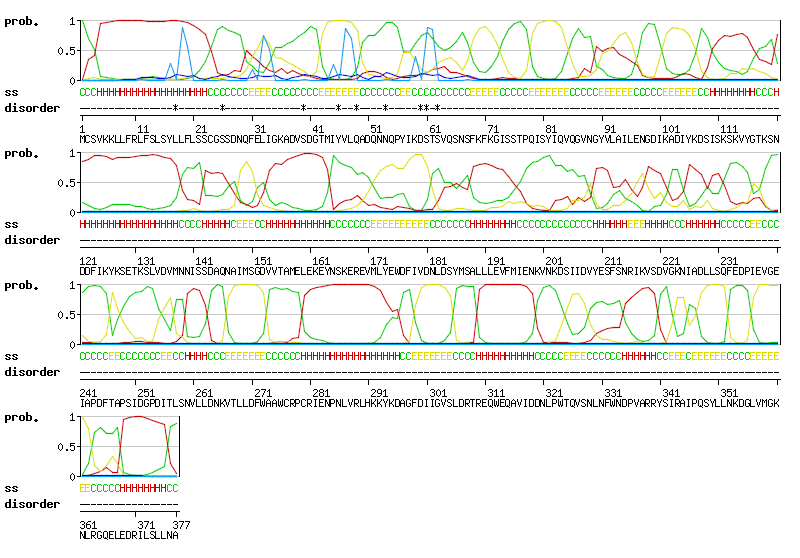

Supplement: Supporting Information S4 [file mmc6.zip › S4_Nilewski_et_al_Predicted_Secondary_Structure/C11.gif]

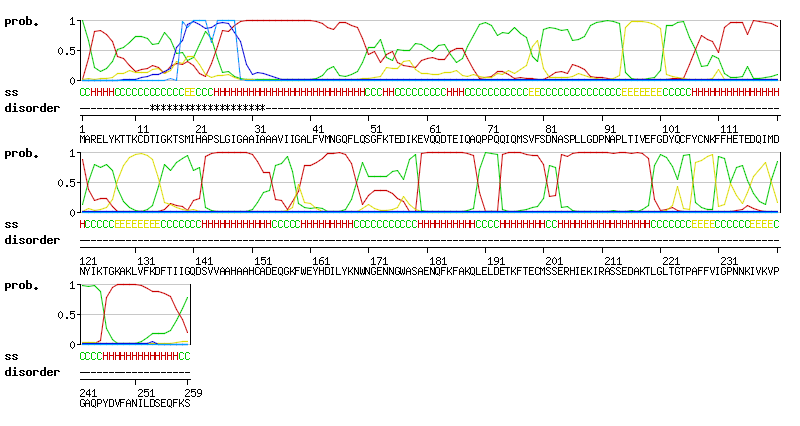

Supplement: Supporting Information S4 [file mmc6.zip › S4_Nilewski_et_al_Predicted_Secondary_Structure/C2.gif]

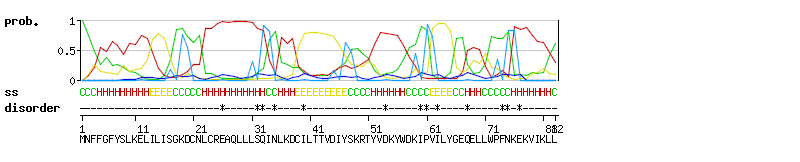

Supplement: Supporting Information S4 [file mmc6.zip › S4_Nilewski_et_al_Predicted_Secondary_Structure/B6.gif]

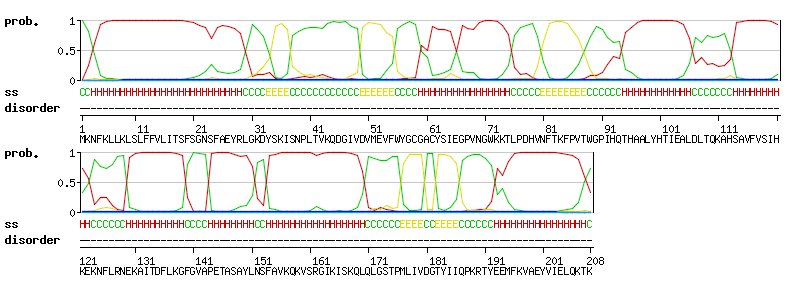

Supplement: Supporting Information S4 [file mmc6.zip › S4_Nilewski_et_al_Predicted_Secondary_Structure/O1.gif]

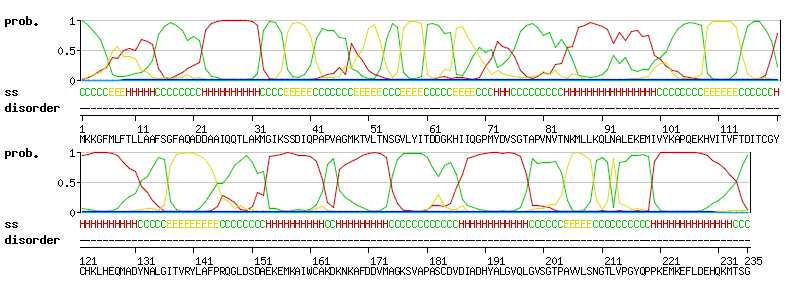

Supplement: Supporting Information S4 [file mmc6.zip › S4_Nilewski_et_al_Predicted_Secondary_Structure/DsbC.gif]

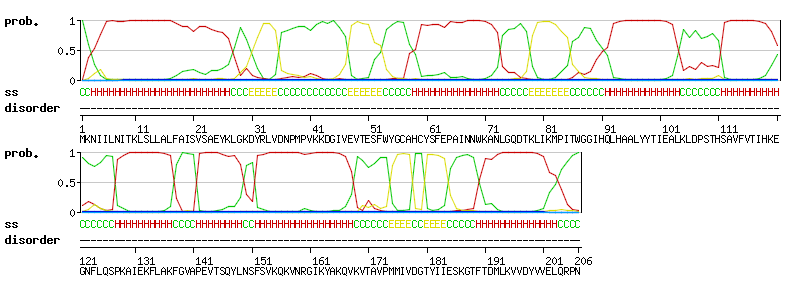

Supplement: Supporting Information S4 [file mmc6.zip › S4_Nilewski_et_al_Predicted_Secondary_Structure/B7.gif]

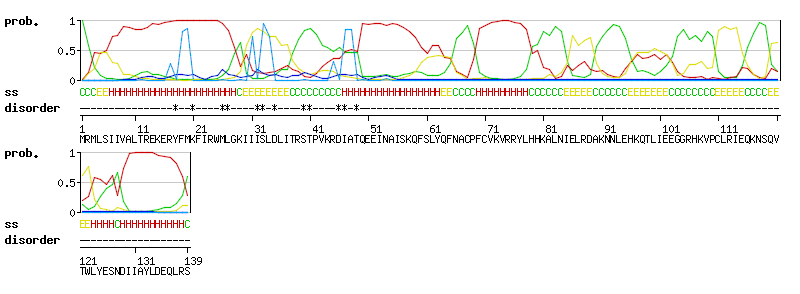

Supplement: Supporting Information S4 [file mmc6.zip › S4_Nilewski_et_al_Predicted_Secondary_Structure/C3.gif]

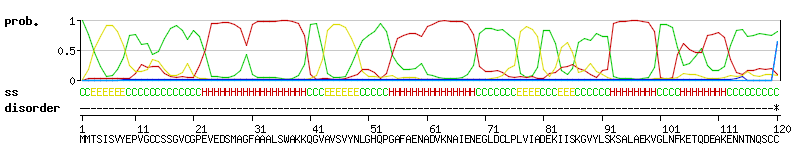

Supplement: Supporting Information S4 [file mmc6.zip › S4_Nilewski_et_al_Predicted_Secondary_Structure/A12.gif]

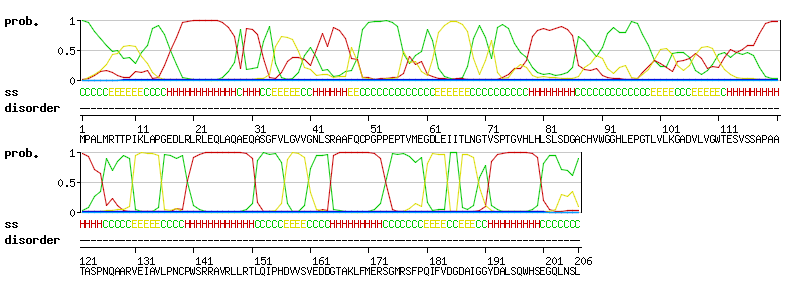

Supplement: Supporting Information S4 [file mmc6.zip › S4_Nilewski_et_al_Predicted_Secondary_Structure/C10.gif]

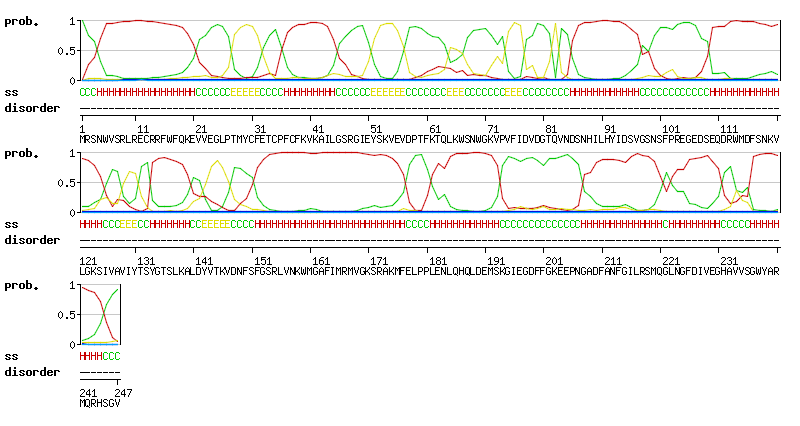

Supplement: Supporting Information S4 [file mmc6.zip › S4_Nilewski_et_al_Predicted_Secondary_Structure/C7.gif]

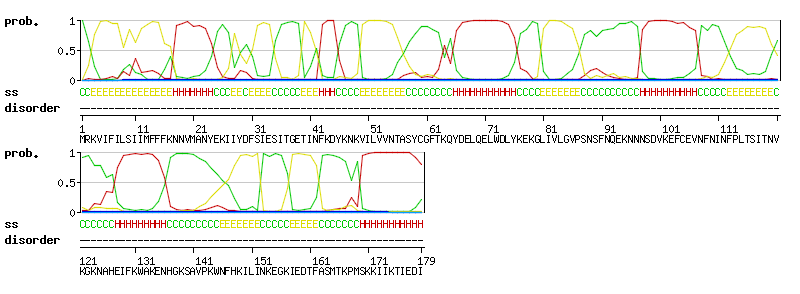

Supplement: Supporting Information S4 [file mmc6.zip › S4_Nilewski_et_al_Predicted_Secondary_Structure/B3.gif]

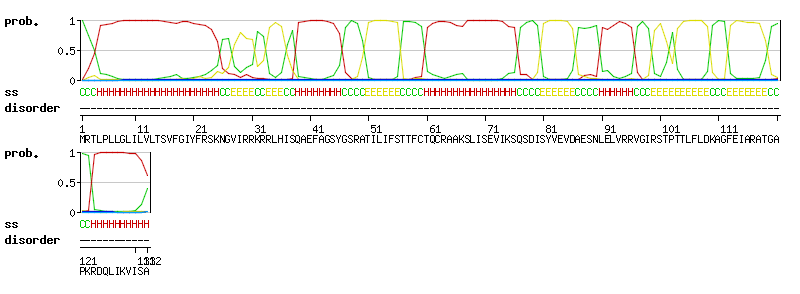

Supplement: Supporting Information S4 [file mmc6.zip › S4_Nilewski_et_al_Predicted_Secondary_Structure/G10.gif]

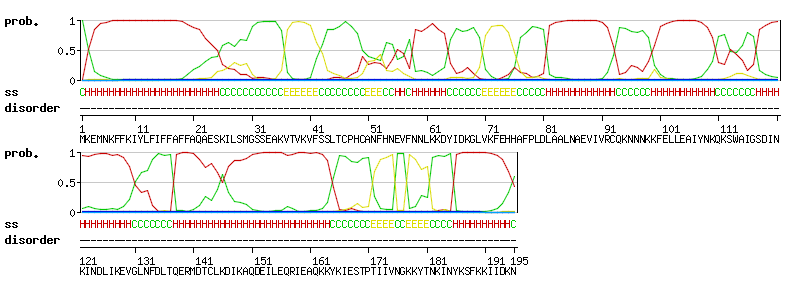

Supplement: Supporting Information S4 [file mmc6.zip › S4_Nilewski_et_al_Predicted_Secondary_Structure/E12.gif]

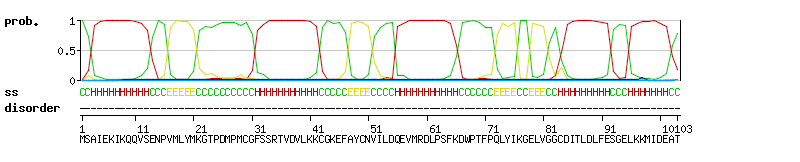

Supplement: Supporting Information S4 [file mmc6.zip › S4_Nilewski_et_al_Predicted_Secondary_Structure/G11.gif]

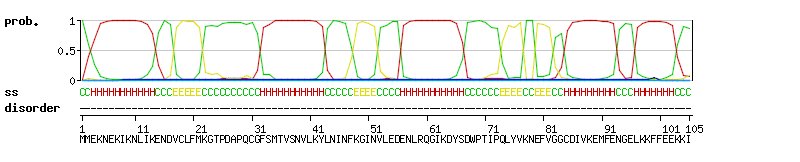

Supplement: Supporting Information S4 [file mmc6.zip › S4_Nilewski_et_al_Predicted_Secondary_Structure/B2.gif]

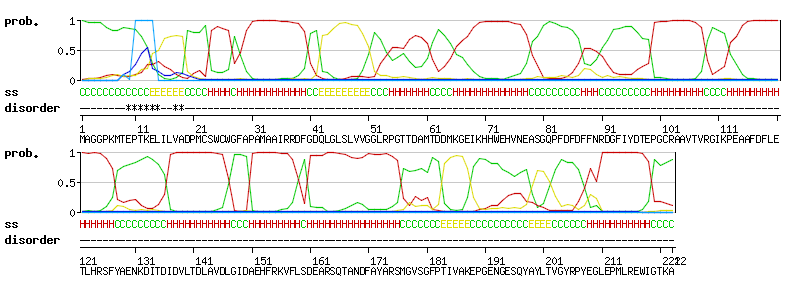

Supplement: Supporting Information S4 [file mmc6.zip › S4_Nilewski_et_al_Predicted_Secondary_Structure/C6.gif]

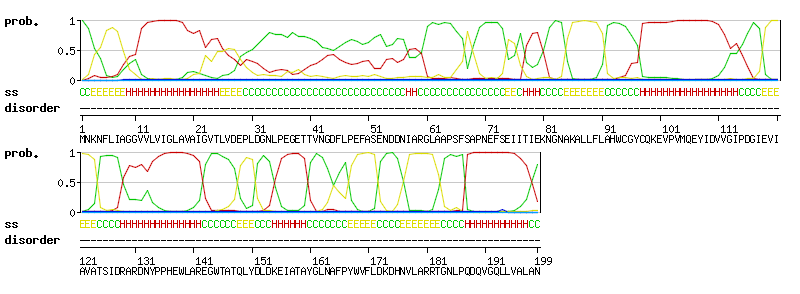

Supplement: Supporting Information S4 [file mmc6.zip › S4_Nilewski_et_al_Predicted_Secondary_Structure/C4.gif]

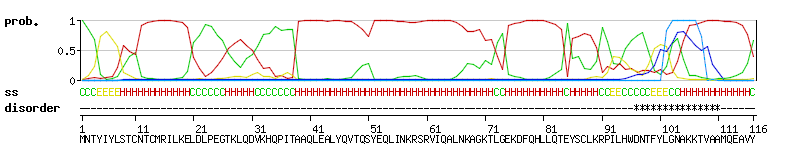

Supplement: Supporting Information S4 [file mmc6.zip › S4_Nilewski_et_al_Predicted_Secondary_Structure/E11.gif]

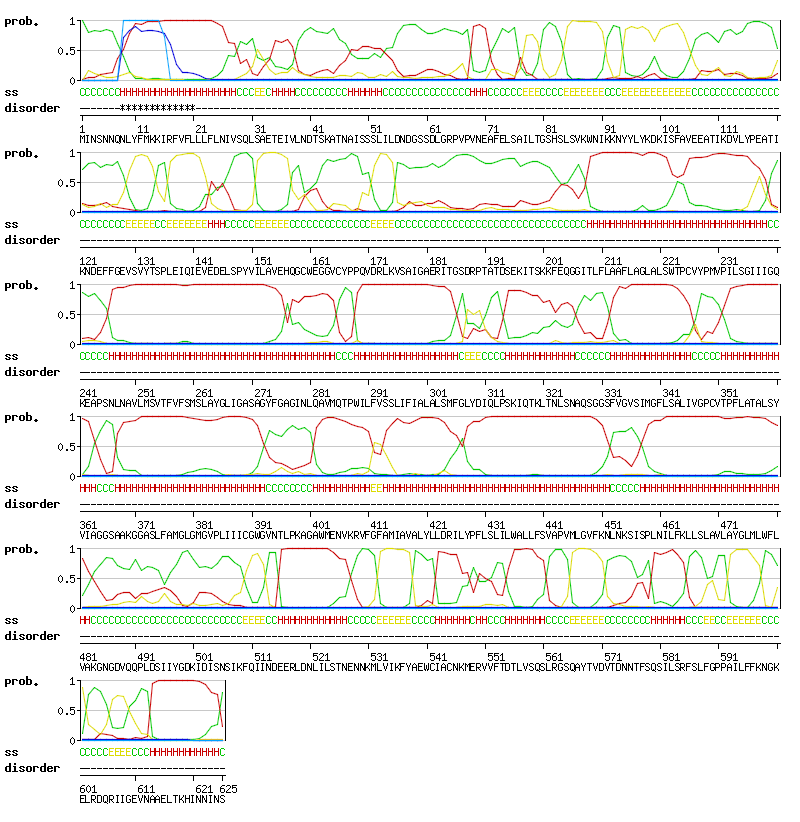

Supplement: Supporting Information S4 [file mmc6.zip › S4_Nilewski_et_al_Predicted_Secondary_Structure/E10.gif]

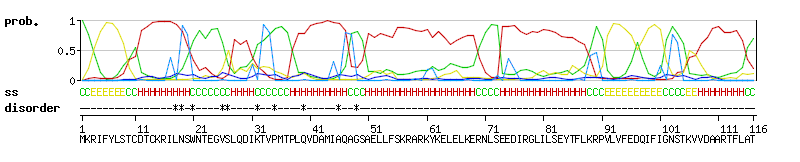

Supplement: Supporting Information S4 [file mmc6.zip › S4_Nilewski_et_al_Predicted_Secondary_Structure/G12.gif]

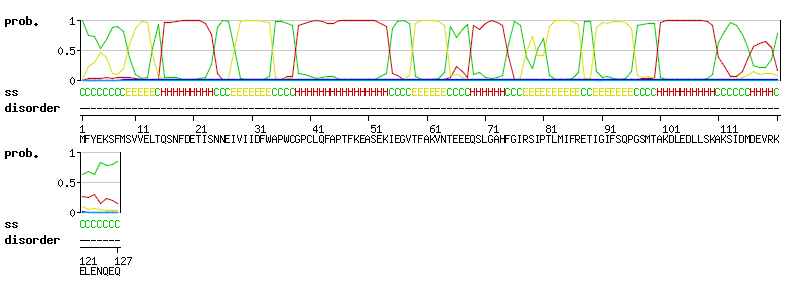

Supplement: Supporting Information S4 [file mmc6.zip › S4_Nilewski_et_al_Predicted_Secondary_Structure/C5.gif]

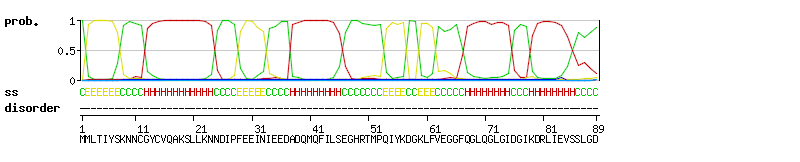

Supplement: Supporting Information S4 [file mmc6.zip › S4_Nilewski_et_al_Predicted_Secondary_Structure/B1.gif]

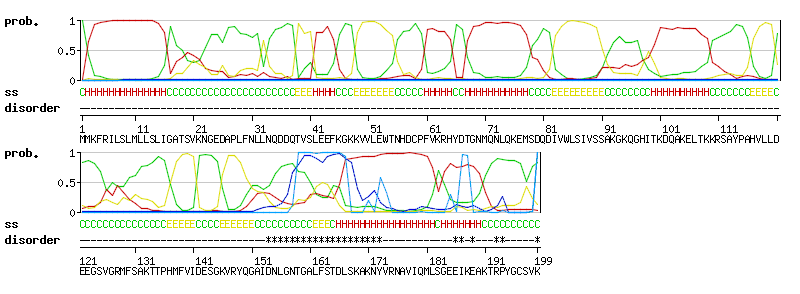

Supplement: Supporting Information S4 [file mmc6.zip › S4_Nilewski_et_al_Predicted_Secondary_Structure/C8.gif]

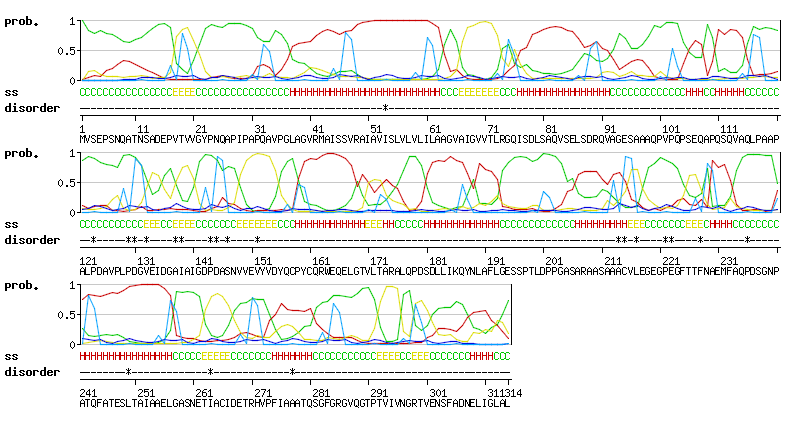

Supplement: Supporting Information S4 [file mmc6.zip › S4_Nilewski_et_al_Predicted_Secondary_Structure/C9.gif]

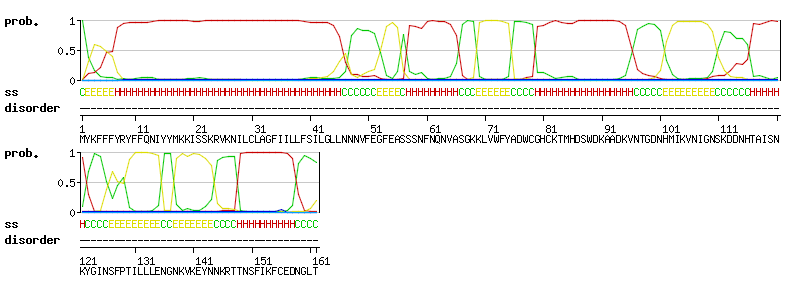

Supplement: Supporting Information S4 [file mmc6.zip › S4_Nilewski_et_al_Predicted_Secondary_Structure/A3.gif]

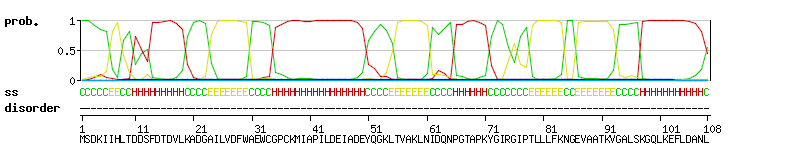

Supplement: Supporting Information S4 [file mmc6.zip › S4_Nilewski_et_al_Predicted_Secondary_Structure/TrxA.gif]

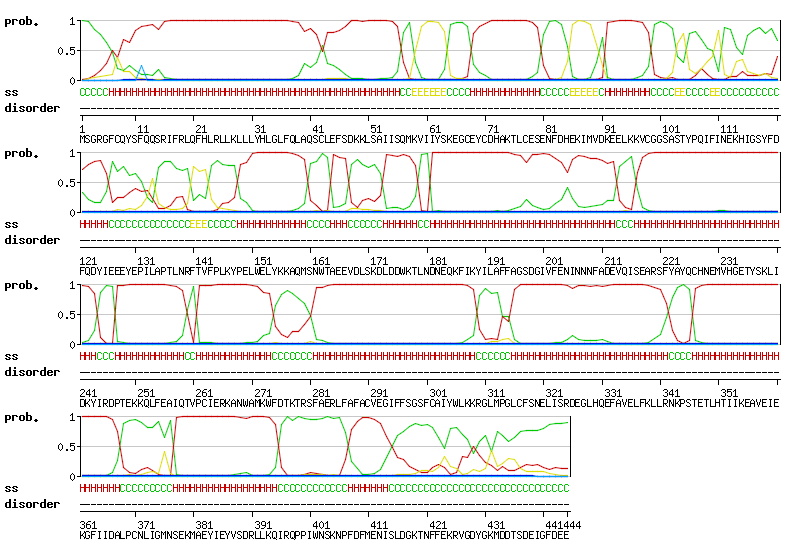

Supplement: Supporting Information S4 [file mmc6.zip › S4_Nilewski_et_al_Predicted_Secondary_Structure/A2.gif]

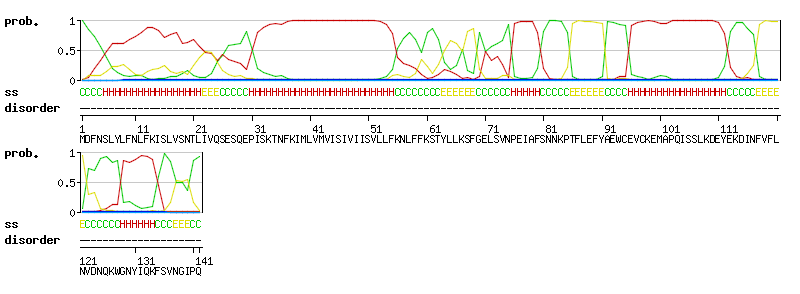

Supplement: Supporting Information S4 [file mmc6.zip › S4_Nilewski_et_al_Predicted_Secondary_Structure/A6.gif]

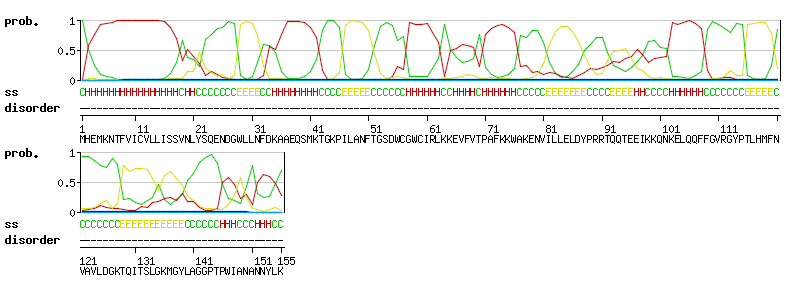

Supplement: Supporting Information S4 [file mmc6.zip › S4_Nilewski_et_al_Predicted_Secondary_Structure/A7.gif]

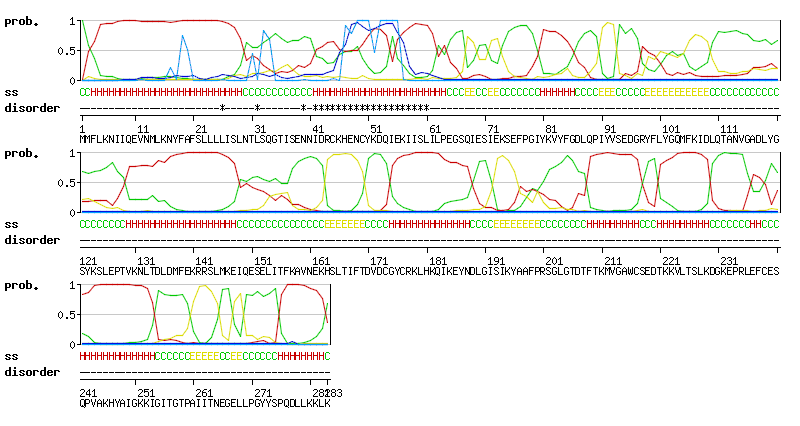

Supplement: Supporting Information S4 [file mmc6.zip › S4_Nilewski_et_al_Predicted_Secondary_Structure/A5.gif]

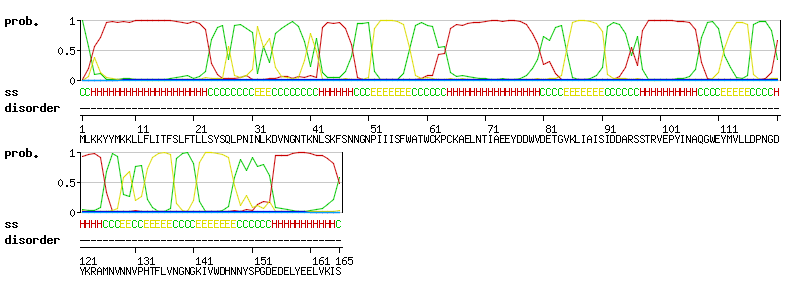

Supplement: Supporting Information S4 [file mmc6.zip › S4_Nilewski_et_al_Predicted_Secondary_Structure/B9.gif]

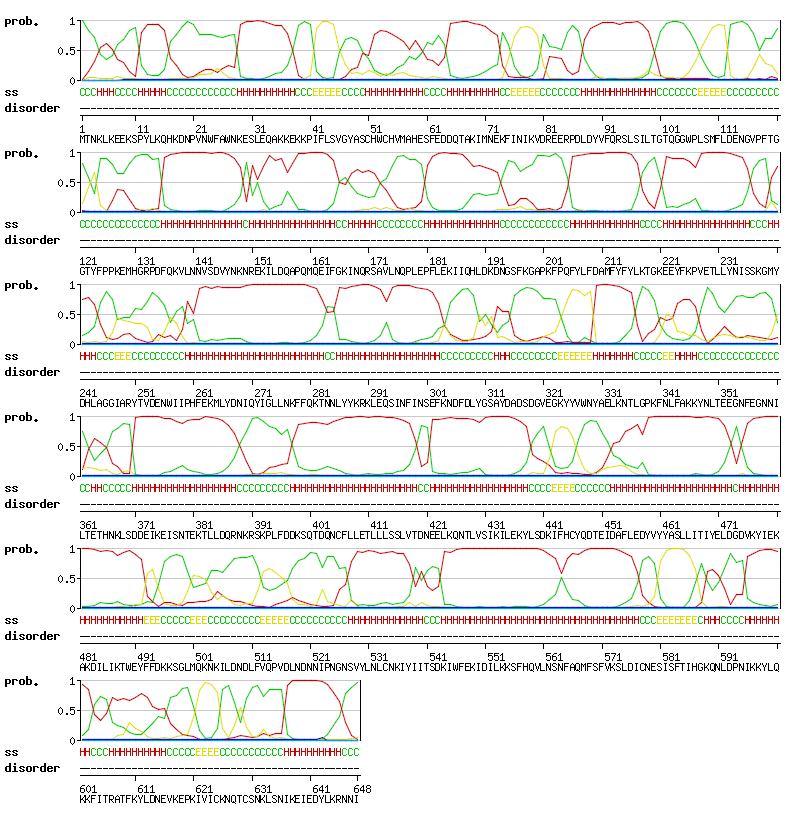

Supplement: Supporting Information S4 [file mmc6.zip › S4_Nilewski_et_al_Predicted_Secondary_Structure/B8.gif]

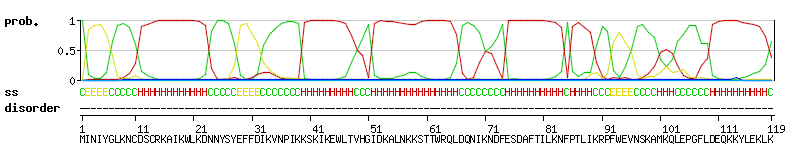

Supplement: Supporting Information S4 [file mmc6.zip › S4_Nilewski_et_al_Predicted_Secondary_Structure/A4.gif]

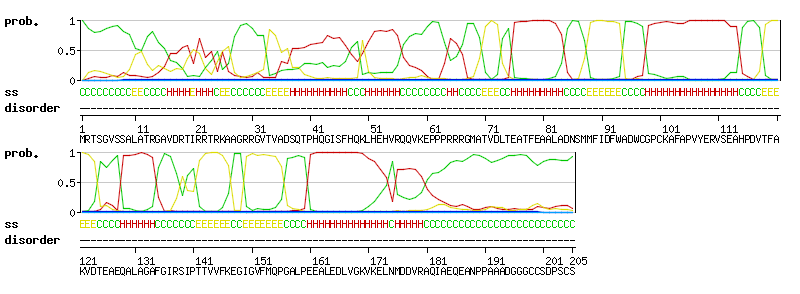

Supplement: Supporting Information S4 [file mmc6.zip › S4_Nilewski_et_al_Predicted_Secondary_Structure/R2.gif]

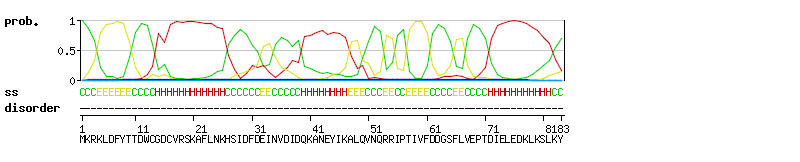

Supplement: Supporting Information S4 [file mmc6.zip › S4_Nilewski_et_al_Predicted_Secondary_Structure/F7.gif]

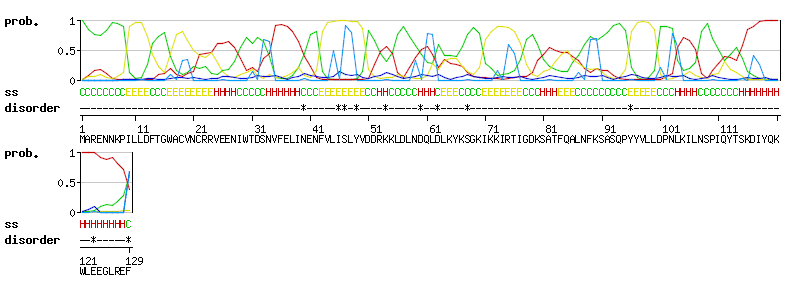

Supplement: Supporting Information S4 [file mmc6.zip › S4_Nilewski_et_al_Predicted_Secondary_Structure/G3.gif]

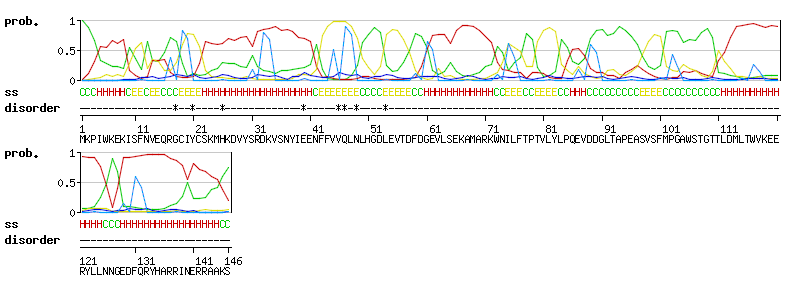

Supplement: Supporting Information S4 [file mmc6.zip › S4_Nilewski_et_al_Predicted_Secondary_Structure/H12.gif]

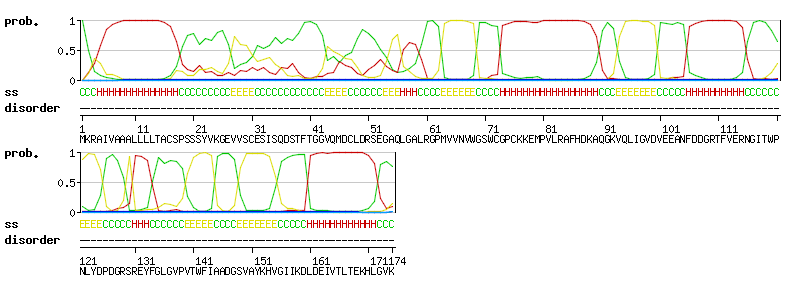

Supplement: Supporting Information S4 [file mmc6.zip › S4_Nilewski_et_al_Predicted_Secondary_Structure/G2.gif]

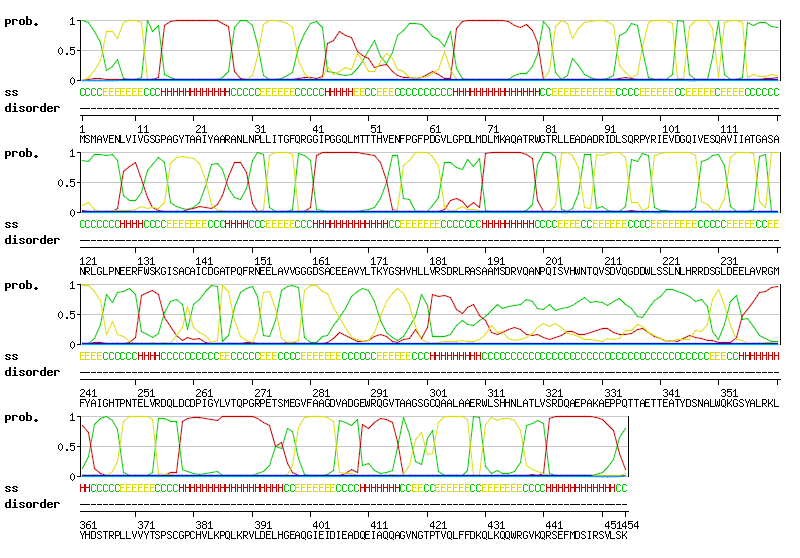

Supplement: Supporting Information S4 [file mmc6.zip › S4_Nilewski_et_al_Predicted_Secondary_Structure/F6.gif]

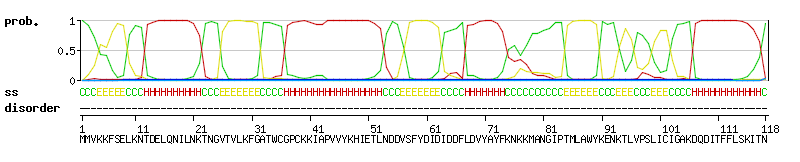

Supplement: Supporting Information S4 [file mmc6.zip › S4_Nilewski_et_al_Predicted_Secondary_Structure/R1.gif]

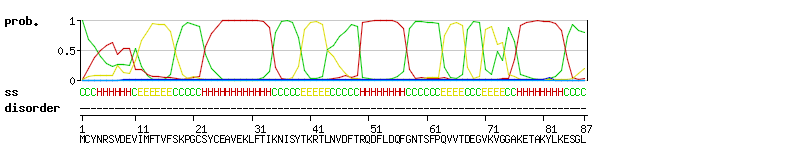

Supplement: Supporting Information S4 [file mmc6.zip › S4_Nilewski_et_al_Predicted_Secondary_Structure/E8.gif]

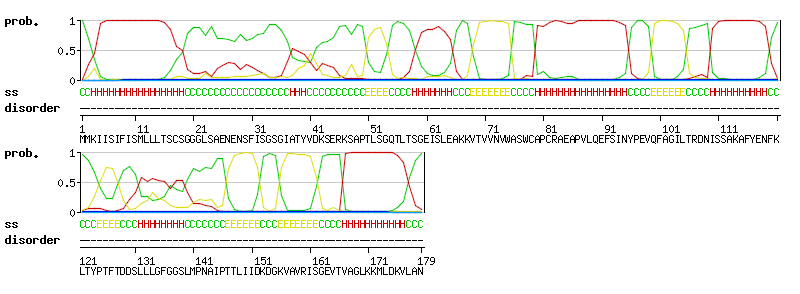

Supplement: Supporting Information S4 [file mmc6.zip › S4_Nilewski_et_al_Predicted_Secondary_Structure/F4.gif]

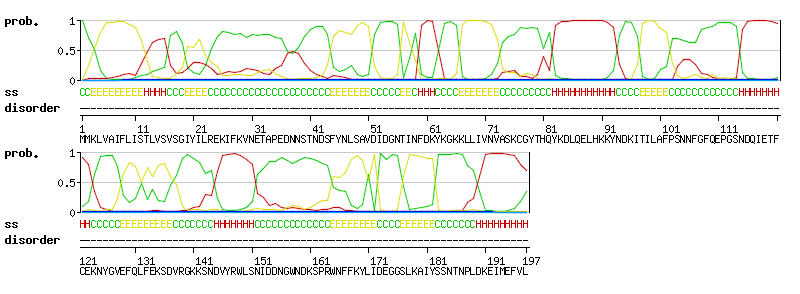

Supplement: Supporting Information S4 [file mmc6.zip › S4_Nilewski_et_al_Predicted_Secondary_Structure/H10.gif]

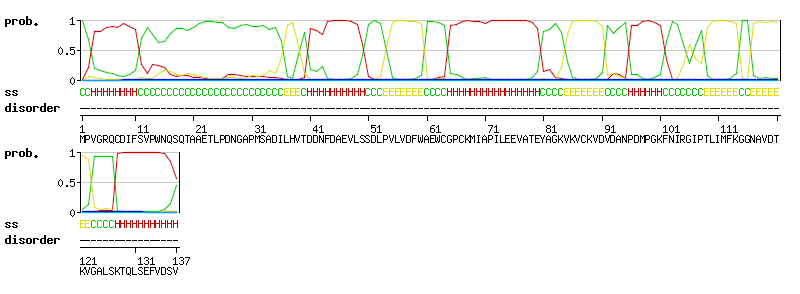

Supplement: Supporting Information S4 [file mmc6.zip › S4_Nilewski_et_al_Predicted_Secondary_Structure/H11.gif]

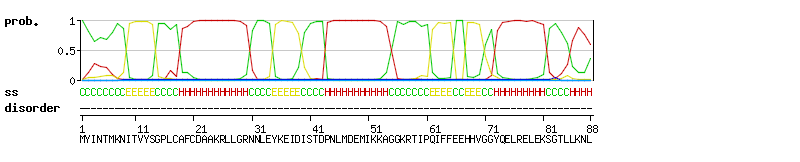

Supplement: Supporting Information S4 [file mmc6.zip › S4_Nilewski_et_al_Predicted_Secondary_Structure/F5.gif]

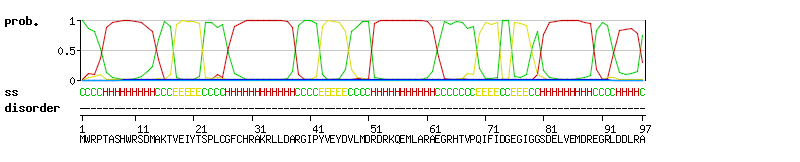

Supplement: Supporting Information S4 [file mmc6.zip › S4_Nilewski_et_al_Predicted_Secondary_Structure/G1.gif]

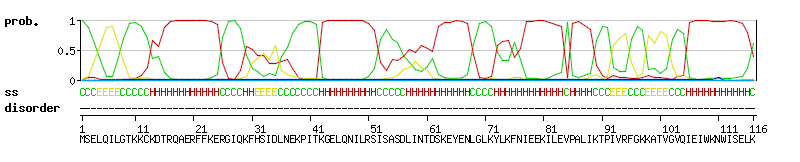

Supplement: Supporting Information S4 [file mmc6.zip › S4_Nilewski_et_al_Predicted_Secondary_Structure/E9.gif]

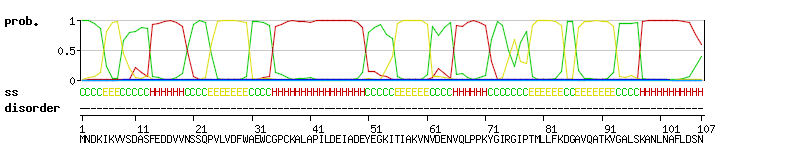

Supplement: Supporting Information S4 [file mmc6.zip › S4_Nilewski_et_al_Predicted_Secondary_Structure/G5.gif]

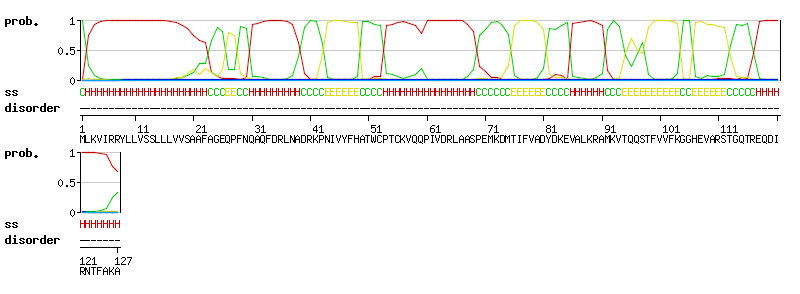

Supplement: Supporting Information S4 [file mmc6.zip › S4_Nilewski_et_al_Predicted_Secondary_Structure/F1.gif]

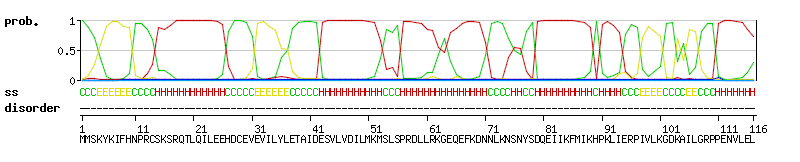

Supplement: Supporting Information S4 [file mmc6.zip › S4_Nilewski_et_al_Predicted_Secondary_Structure/D9.gif]

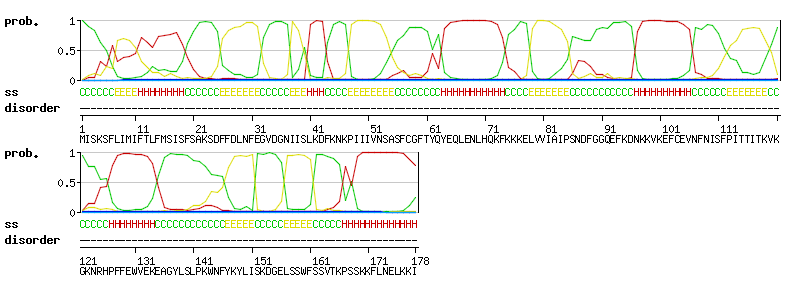

Supplement: Supporting Information S4 [file mmc6.zip › S4_Nilewski_et_al_Predicted_Secondary_Structure/D8.gif]

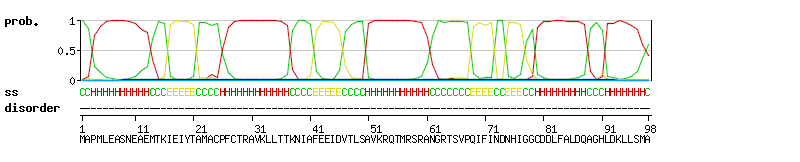

Supplement: Supporting Information S4 [file mmc6.zip › S4_Nilewski_et_al_Predicted_Secondary_Structure/G4.gif]

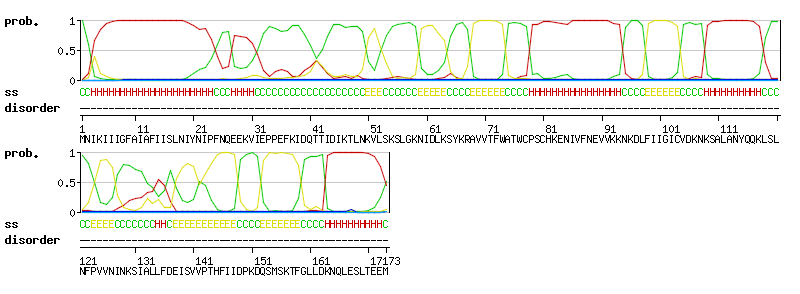

Supplement: Supporting Information S4 [file mmc6.zip › S4_Nilewski_et_al_Predicted_Secondary_Structure/G6.gif]

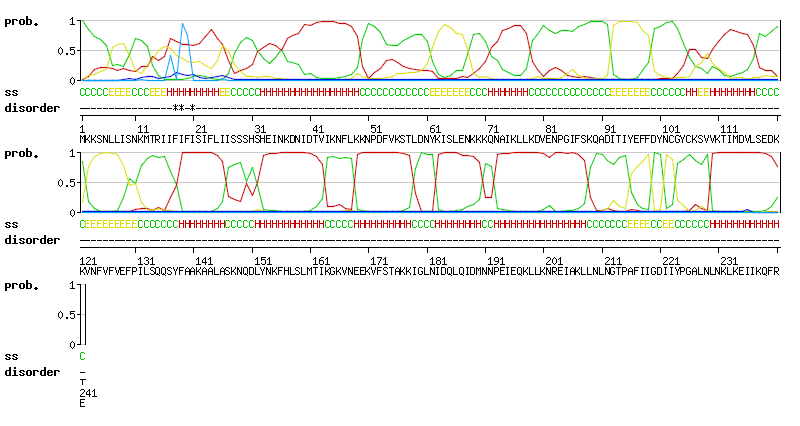

Supplement: Supporting Information S4 [file mmc6.zip › S4_Nilewski_et_al_Predicted_Secondary_Structure/H9.gif]

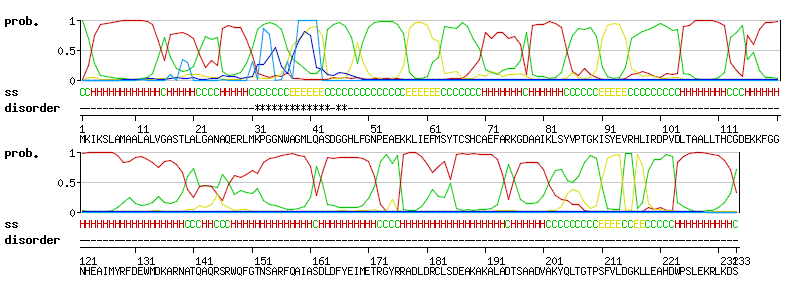

Supplement: Supporting Information S4 [file mmc6.zip › S4_Nilewski_et_al_Predicted_Secondary_Structure/H8.gif]

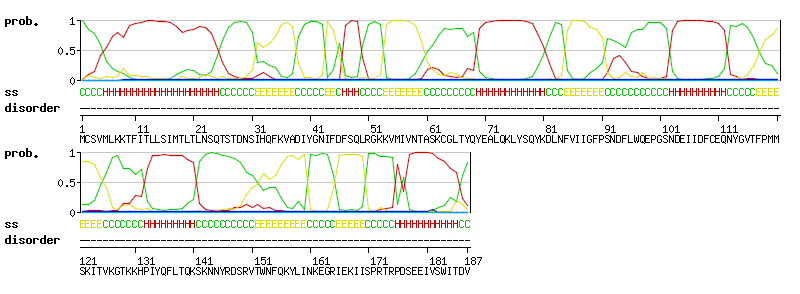

Supplement: Supporting Information S4 [file mmc6.zip › S4_Nilewski_et_al_Predicted_Secondary_Structure/G7.gif]
