# Supplementary material for: Functional metagenomics of the thioredoxin superfamily
Source: J Biol Chem. 2021 Jan 14;296:100247. doi: 10.1074/jbc.RA120.016350 (PMC7949104; doi:10.1074/jbc.RA120.016350)
Supplement: Supplemental Figure S2 [file mmc2.pdf]

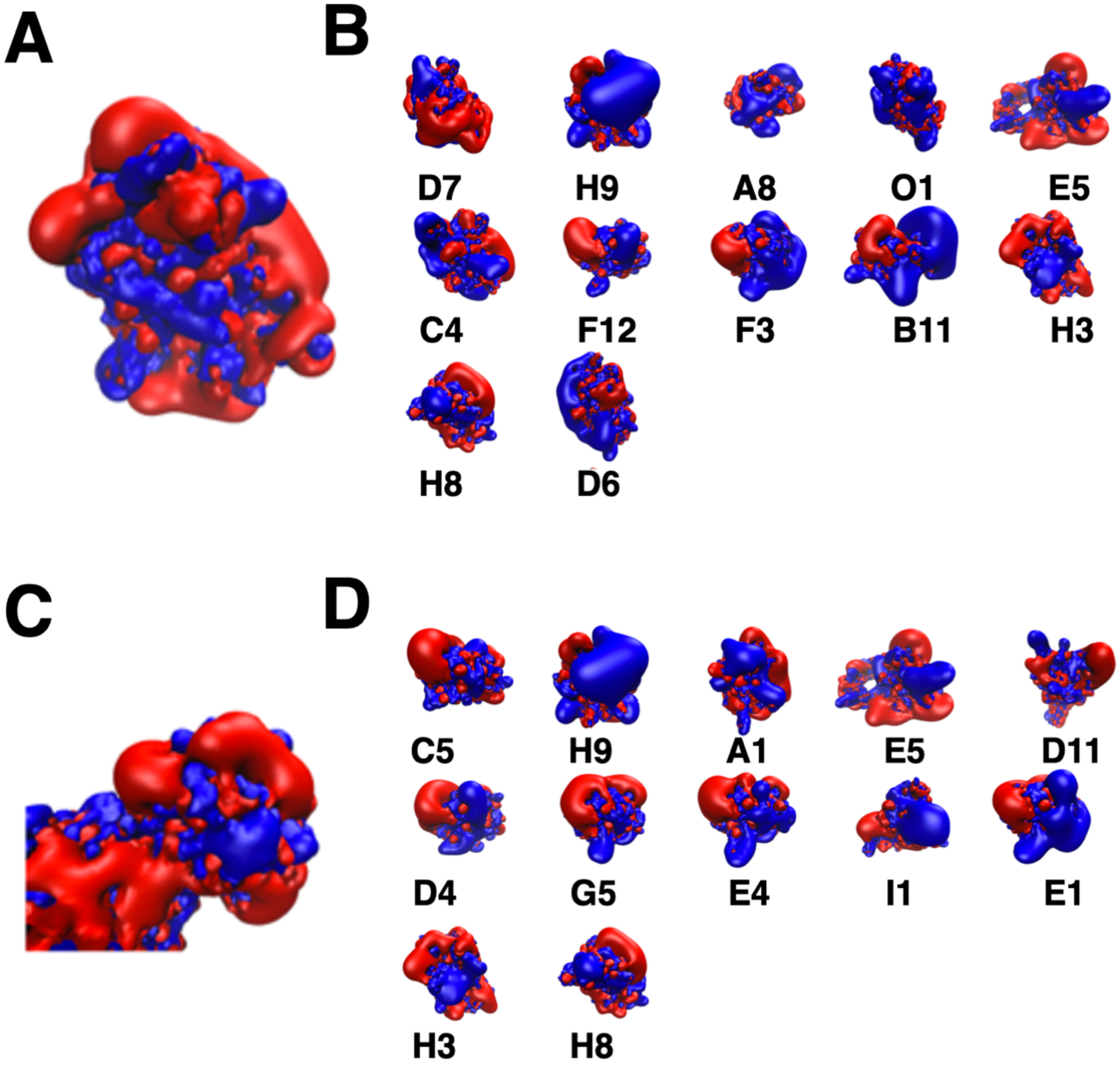

Figure S2: Predicted isoelectric surface charges of thioredoxin superfamily members that rescue Δ*dsbA* and Δ*dsbC*/*mdoG* mutant phenotypes. (A) Isoelectric surface charge of *E. coli* DsbA. (B) Isosurfaces of the electrostatic potential based on predicted structures of metagenomic oxidases. Orientation of the proteins is identical based on the structural alignment with *E. coli* DsbA's structure. (C) Isoelectric surface charge of *E. coli* DsbC. (D) Isosurfaces of the electrostatic potential based on predicted structures of metagenomic isomerases. Orientation of the proteins is identical based on the structural alignment with *E. coli* DsbC's structure.
