# Supplementary figures and images for: Functional metagenomics of the thioredoxin superfamily (part 2 of 2)
Source: J Biol Chem. 2021 Jan 14;296:100247. doi: 10.1074/jbc.RA120.016350 (PMC7949104; doi:10.1074/jbc.RA120.016350)

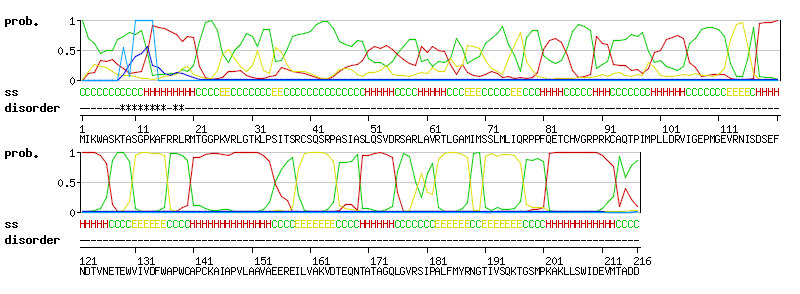

Supplement: Supporting Information S4 [file mmc6.zip › S4_Nilewski_et_al_Predicted_Secondary_Structure/F3.gif]
